# A novel combination of niraparib and anlotinib in platinum-resistant ovarian cancer: Efficacy and safety results from the phase II, multi-center ANNIE study

**DOI:** 10.1016/j.eclinm.2022.101767

**Published:** 2022-11-30

**Authors:** Guochen Liu, Yanling Feng, Jing Li, Ting Deng, Aijun Yin, Lei Yan, Min Zheng, Ying Xiong, Jundong Li, Yongwen Huang, Chuyao Zhang, He Huang, Ting Wan, Qidan Huang, An Lin, Jie Jiang, Beihua Kong, Jihong Liu

**Affiliations:** a Sun Yat-Sen University Cancer Center, State Key Laboratory of Oncology in South China, Collaborative Innovation Center for Cancer Medicine, 651 Dongfeng Road East, Guangzhou, 510060, China; b Department of Obstetrics and Gynecology, Qilu Hospital, Shandong University, 107 West Wenhua Road, Jinan, 250012, China; c The First Affiliated Hospital of Jinan University, 613 Huangpu Avenue West, Guangzhou, 510630, China; d Fujian Provincial Cancer Hospital, No. 91, Fengpanma Road, Fuma Road, Fuzhou, 350014, China

**Keywords:** Platinum-resistant, Ovarian neoplasia, Niraparib, Anlotinib

## Abstract

**Background:**

Patients with platinum-resistant recurrent ovarian cancer (PROC) face poor prognosis and limited treatment options. Single-agent antiangiogenics and poly (ADP-ribose) polymerase (PARP) inhibitors both show some activities in platinum-resistant diseases. The ANNIE study aimed to evaluate the efficacy and safety of the novel combination of the PARP inhibitor niraparib and the antiangiogenic anlotinib in patients with PROC.

**Methods:**

ANNIE is a multicentre, single-arm, phase 2 study (ClinicalTrials.gov identifier NCT04376073) conducted at three hospitals in China. Eligible patients had histologically confirmed epithelial ovarian, fallopian tube, or primary peritoneal cancer that recurred within 6 months of last platinum-based chemotherapy. Patients with prior PARP inhibitor exposure were excluded. The enrolled patients received oral niraparib 200 mg or 300 mg (baseline body weight-directed) once daily continuously and anlotinib 10 mg (12 mg before protocol amendment) once daily on days 1–14 of each 21-day cycle until disease progression or intolerable toxicity. The primary endpoint was objective response rate (ORR).

**Findings:**

Between May 22, 2020, and April 22, 2021, 40 patients were enrolled and treated. Thirty-six patients underwent post-baseline tumour assessments. By data cut-off (January 31, 2022), median follow-up was 15.4 months (95% CI 12.6–17.7). Intention-to-treat ORR was 50.0% (95% CI 33.8–66.2), including one complete response and 19 partial responses. Median (95% CI) progression-free survival and overall survival were 9.2 months (7.4–11.9) and 15.3 months (13.9–not evaluable), respectively. Drug-related, grade ≥3 TEAEs were reported in 26 (68%) patients. There were no treatment-related deaths.

**Interpretation:**

Niraparib plus anlotinib showed promising antitumour activity in patients with PROC. This oral combination warrants further investigation as a potential novel, convenient treatment option for patients with PROC.

**Funding:**

Zai Lab (Shanghai) Co., Ltd; Jiangsu Chia Tai-Tianqing Pharmaceutical Co., Ltd; the 10.13039/501100001809National Natural Science Foundation of China (No. 82102783).

**Research in context**

**Evidence before this study**

The combination of antiangiogenics and PARP inhibitors has been being explored as a "chemo-free" treatment option for patients with platinum-resistant recurrent ovarian cancer (PROC). We searched PubMed and Web of Science up to February 2022 with the search terms (“PARP inhibitors” OR “PARPi” OR “poly ADP-ribose polymerase inhibitors”) AND (“antiangiogenics”) AND (“Platinum-Resistant Ovarian Cancer” OR “PROC” OR “platinum-resistant recurrent ovarian cancer”). Cediranib–olaparib is the only such combination identified. However, the efficacy and safety of this combination are not satisfactory. Niraparib and anlotinib have distinct metabolic pathways and thus minimal drug–drug interaction, conducive for the safe use of these drugs combined.

**Added value of this study**

ANNIE is the first study to investigate the niraparib–anlotinib combination in PROC. The efficacy data and safety profile of the combination of niraparib and anlotinib in the ANNIE study demonstrate a favourable risk–benefit profile in platinum-resistant diseases. The ANNIE study provides initial evidence that the combination might improve the objective response rate and progression-free survival in patients with PROC.

**Implications of all the available evidence**

Combinations of antiangiogenics and PARP inhibitors represent an attractive target for developing novel treatment options for platinum-resistant recurrent ovarian cancer. The niraparib–anlotinib combination may provide a potential treatment option for such patients, and our findings warrant validation in future larger trials.

## Introduction

Epithelial ovarian cancer (including epithelial ovarian, fallopian tube, and primary peritoneal cancers) is one of the leading causes of female cancer death among all cancer types.^1^ Due to the lack of effective screening methods, more than 70% of patients are diagnosed at an advanced stage.^2^ Despite initial responses to platinum- and taxane-based chemotherapies, most patients will relapse within 2 years.^3^ With the gradual shortening of the between-recurrence interval, these patients would progress from platinum-sensitive recurrence to platinum-resistant recurrence eventually.

For patients with platinum-resistant recurrent ovarian cancer (PROC), effective treatment options are limited. Standard nonplatinum-based chemotherapies such as weekly paclitaxel, pegylated liposomal doxorubicin, and topotecan yield objective response rates (ORR) of 10%–30% and progression-free survival (PFS) of 2–5 months.^4^^,^^5^ Among the other currently approved treatment options, the antiangiogenic bevacizumab significantly improved the PFS and ORR when added to chemotherapies, but with an increased incidence of adverse events.^6^ The PARP inhibitors olaparib and niraparib can be used in platinum-resistant patients after ≥2 or ≥3 prior lines of therapy, but subject to the presence of *BRCA* mutations or homologous recombination deficiency (HRD).^4^ Moreover, there is also a need for better tolerated regimens. Many platinum-resistant patients would have received multiple prior lines of chemotherapy, with compromised health status and unable to tolerate further chemotherapy at sufficient doses or frequencies. The intravenous administration of bevacizumab and most chemotherapies also inconveniences patients and may affect quality of life.

Antiangiogenics and PARP inhibitors both play prominent roles in ovarian cancer treatment, and research into their combinations as novel treatment options has been gaining momentum.^7^ In cells with HRD, PARP inhibition causes accumulation of double-strand breaks that cannot be efficiently repaired, leading to increased genomic instability and ultimately cell death.^7^ Preclinical studies suggest that antiangiogenics can induce HRD through microenvironment hypoxia and downregulation of homologous recombination repair genes, thereby sensitizing tumour cells to PARP inhibitors.^8^^,^^9^ Conversely, PARP1 may be implicated in angiogenesis^10^^,^^11^ and PARP inhibitors may thus bring additional antiangiogenic effects. Such is the mechanistic basis for the potential synergy between antiangiogenics and PARP inhibitors, which has since been demonstrated in platinum-sensitive recurrent ovarian cancer for the cediranib–olaparib and the niraparib–bevacizumab combinations.^12^, ^13^, ^14^ As monotherapies, PARP inhibitors and antiangiogenics targeting vascular endothelial growth factor (VEGF) and the VEGF receptor (VEGFR) signalling pathways have both demonstrated antitumour activity in platinum-resistant and/or recurrent ovarian cancers.^7^ It is of interest to investigate whether combining these two classes of drug would bring enhanced efficacy for treating PROC, where cediranib–olaparib is the only combination extensively evaluated thus far.^15^, ^16^, ^17^

Some medical institutions in China had seen anecdotal clinical use (with full informed consent) of the PARP inhibitor niraparib plus the antiangiogenic anlotinib in patients with PROC, who had failed multi-line chemotherapies and lacked any other treatment options. Observations from such patients yielded preliminary evidence for clinical efficacy, suggesting that the niraparib–anlotinib combination may be a new treatment option worth exploring for this difficult-to-treat patient population.

Niraparib is a highly selective inhibitor of PARP 1/2 approved as maintenance treatment for newly diagnosed or platinum-sensitive recurrent ovarian cancer, and as fourth-or-later-line treatment regardless of platinum sensitivity in the presence of HRD.^18^ Niraparib demonstrated certain clinical benefits even in patients with HRD-negative advanced ovarian cancer, in both early- and late-line settings.^19^, ^20^, ^21^ In the AVANOVA study, the combination of niraparib and bevacizumab showed better efficacy than niraparib alone with tolerable adverse effects in patients with platinum-sensitive recurrent ovarian cancer.^13^

Anlotinib is an oral small-molecule receptor tyrosine kinase inhibitor (TKI) that targets VEGFR1/2/3, c-Kit, PDGFR-α, and fibroblast growth factor receptors (FGFR1/2/3), with a broad spectrum of inhibitory effects on tumour angiogenesis and growth.^22^ Anlotinib is approved in China as salvage treatment for several cancer types including lung cancer and soft tissue sarcoma. Evidence is emerging for the efficacy of anlotinib in platinum-resistant ovarian cancer.^23^ Niraparib and anlotinib have distinct metabolic pathways and thus minimal drug–drug interaction, conducive for the safe use of these drugs in combination. In this study, we evaluated the safety and activity of the niraparib–anlotinib combination in patients with PROC.

## Methods

### Study design and participants

The ANNIE study (ClinicalTrials.gov identifier NCT04376073) was a single-arm, open-label, phase 2 trial conducted at three hospitals in China. The study protocol was approved by the institutional review board at each participating site, and written informed consent was obtained from each participating patient. The Sun Yat-Sen University Cancer Center Monitoring Board provided oversight of the study.

Eligible patients were aged 18–70 years old with histologically confirmed ovarian epithelial, fallopian tube, or primary peritoneal cancer that recurred in less than 6 months after the last administered platinum-based therapy. Patients were required to have measurable disease according to the Response Evaluation Criteria in Solid Tumours (RECIST) version 1.1. Patients must have germline (g*BRCA*) or tumour (t*BRCA*) *BRCA* test results available prior to enrolment, and patients of any *BRCA* or homologous recombination status were eligible. Patients had to have a life expectancy of longer than 16 weeks, an Eastern Cooperative Oncology Group performance status score of 0 or 1, and adequate organ functions. Patients with prior antiangiogenic treatment were eligible, while patients with prior exposure to PARP inhibitors were excluded. There was no restriction on the number of platinum-based chemotherapies received. Patients with the following conditions were excluded: allergy to study medication; symptomatic brain metastases; major surgery procedure within three weeks; any cancer other than ovarian cancer within two years; uncontrollable cardiovascular diseases.

### Procedures

Eligible patients received niraparib plus anlotinib treatment in consecutive 21-day cycles. Anlotinib was administered orally on days 1–14 of each cycle at 12 mg or 10 mg once a day (QD) (In the interim analysis for the first 23 patients, toxicities were observed mainly in the form of hand-foot syndrome [n = 8, with n = 2 for grade ≥3] and hypertension [n = 7, with n = 1 for grade ≥3]. Therefore, a protocol amendment on November 25, 2020 reduced the starting dose of anlotinib from 12 mg QD to 10 mg QD). Niraparib was administered orally and daily at 300 mg QD (if baseline bodyweight ≥77 kg) or 200 mg QD (if baseline bodyweight <77 kg). The first administration of the study medication must be >4 weeks after the end of the previous antitumour treatment (>6 weeks if the previous regimen contained mitomycin).

Patients received study treatment until disease progression, unacceptable toxicity, withdrawal of consent, death, or loss to follow-up, whichever occurred first. Dose modification was permitted for managing adverse events, where the doses of anlotinib and niraparib could be reduced or interrupted independently of each other at the investigators’ discretion. The maximal duration permitted of dose interruption was 28 days.

Patients were required to record daily medication, heart rate, blood pressure, and symptoms in a follow-up manual. The study staff contacted all patients by phone weekly, and patients were instructed to contact the study staff immediately in the case of abnormal symptoms. Tests for blood haematology and serum chemistry, urine tests, and electrocardiogram were performed once every three weeks (complete blood count was performed weekly for the first four weeks). Tumour assessment was conducted at baseline and once every three cycles thereafter using computed tomography or magnetic resonance imaging. The objective assessment of disease progression was determined by the Central Radiology Committee according to RECIST 1.1 and reviewed for sensitivity assessment by two independent radiologist experts who were not otherwise involved in the study. Complete or partial responses must be confirmed >4 weeks after the initial record of response. Elevated CA125 alone was not considered disease progression. Upon treatment discontinuation, adverse events were documented for 30 days after the last dose of study medication and patients were followed-up once every 90 days for survival and subsequent antitumour treatment.

### Outcomes

The primary endpoint was ORR (the percentage of patients with confirmed complete or partial responses) as per RECIST 1.1. Secondary efficacy endpoints included PFS, time to response (TTR), duration of response (DOR), platinum-free interval (PFI), and overall survival (OS). Safety endpoints included the incidence, severity, and drug-relatedness of treatment-emergent adverse events (TEAEs).

An exploratory analysis was performed *post-hoc* to investigate the relationship between circulating tumour markers and prognosis. Available archival peripheral blood samples from baseline and post-baseline follow-up visits were analysed at the core facility of Precision Scientific (Beijing, China), where germline DNA extracted from blood cells and circulating cell-free DNA extracted from plasma were sequenced for 561 genes frequently mutated in ovarian cancer and other solid tumours. Molecular tumour burden index was calculated based on the circulating tumour DNA (ctDNA) detected^24^ and analysed for correlation with the tumour size data from corresponding patients and timepoints.

### Statistical analysis

Historically, the response rate with chemotherapy alone for PROC was about 20%.^2^^,^^25^ Assuming a true ORR of 40% with niraparib–anlotinib, a sample size of 36 patients was estimated to provide 80% power to reject the null hypothesis of an ORR ≤20% at a one-sided type-1 error rate of 5%. Assuming a 10% rate of loss to follow-up, a target sample size of 40 patients was planned. An interim analysis was planned at week 24 after the end of enrolment, whereby if the ORR was below 20% the study would be terminated.

ORR was calculated for the intention-to-treat (ITT) population (all patients who received at least one dose of study medication) and the efficacy-evaluable population (patients with results available for at least one post-baseline tumour assessment). Point estimate and two-sided 95% CI of ORR were calculated using the Clopper-Pearson method. For time-to-event endpoints, the median and corresponding two-sided 95% CI were estimated using the Kaplan–Meier method. For post-hoc subgroup analyses, HRs and corresponding 95% CIs were estimated using a Cox model including the stratification factors as covariates. Safety results were analysed in the ITT population and summarized by number and percentage of patients. Statistical analyses were performed using SAS (SAS Institute) version 9.4.

### Role of the funding source

The study was designed by the investigators. Data from the study were collected by investigators, analysed by investigators, and interpreted by the authors. The manuscript was prepared by a medical writer with inputs from the authors. The sponsors were not involved in the design, collection, analysis, and interpretation of the data, manuscript writing, or the decision to submit for publication. JHL and GCL had full access to the data in the study and all authors had final responsibility for the decision to submit for publication.

## Results

### Participants

**Fig. 1:**
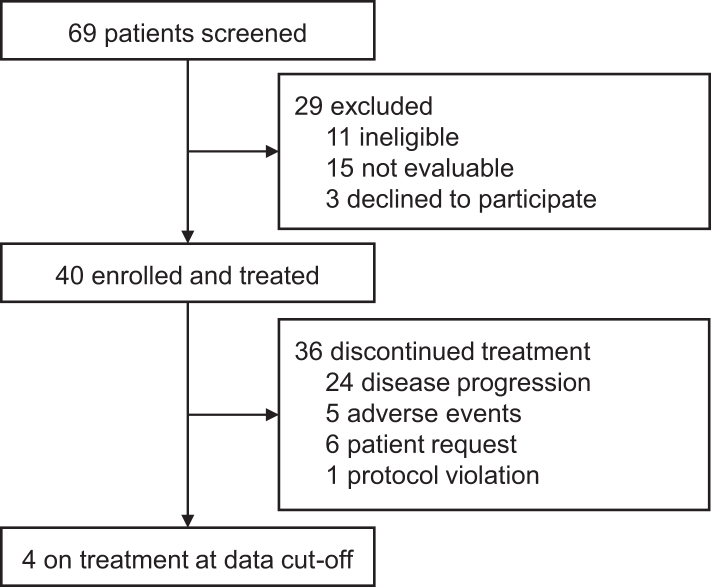
Trial profile.

From May 22, 2020, to April 22, 2021, 40 patients were enrolled from three sites in China (Fig. 1). All 40 patients received niraparib and anlotinib treatment, constituting the ITT population. Only one patient weighing 78 kg received a starting dose of 300 mg of niraparib. The cut-off date for the current analysis was January 31, 2022, and follow-up is ongoing. The median duration of follow-up was 15.4 months (95% CI 12.6–17.7). By data cut-off, four patients were still on treatment. Treatment discontinuations were due to disease progression (n = 24), adverse events (n = 5), patient request (n = 6), and protocol violation (n = 1, received chemotherapy).

Baseline demographic and clinical characteristics are summarized in Table 1. The median age was 54 years (range 37–69). The predominant tumour pathology was high-grade serous adenocarcinoma, and most patients had stage III or IV diseases at the time of diagnosis. The patients had received a median of 4 (range 1–9) lines of chemotherapy. Majority of patients (68%, 27/40) had received prior antiangiogenic treatments, as summarized in Supplementary Table S1. Five patients carried germline *BRCA* mutations (g*BRCA*mut).

**Table 1:** Baseline patient characteristics (N = 40).

Characteristic	Patients
Age, years, median (range)	54 (37–69)
Pathology	
High-grade serous carcinoma	34 (85)
Clear cell carcinoma	2 (5)
Endometrioid carcinoma	2 (5)
High-grade mucinous carcinoma	1 (3)
Low-grade serous carcinoma	1 (3)
FIGO stage at diagnosis	
Ⅰ	1 (3)
Ⅱ	2 (5)
Ⅲ	31 (78)
Ⅳ	6 (15)
Prior lines of chemotherapy, median (range)	4 (1–9)
Received prior antiangiogenic treatment	
Yes	27 (68)
No	13 (33)
ECOG performance status score	
0	25 (63)
1	15 (38)
Germline *BRCA* status	
mutation	5 (13)
wildtype	35 (88)

Note: Data are presented as No. (%) unless indicated otherwise. FIGO, International Federation of Gynecology and Obstetrics; ECOG, Eastern Cooperative Oncology Group.

### Efficacy

**Fig. 2:**
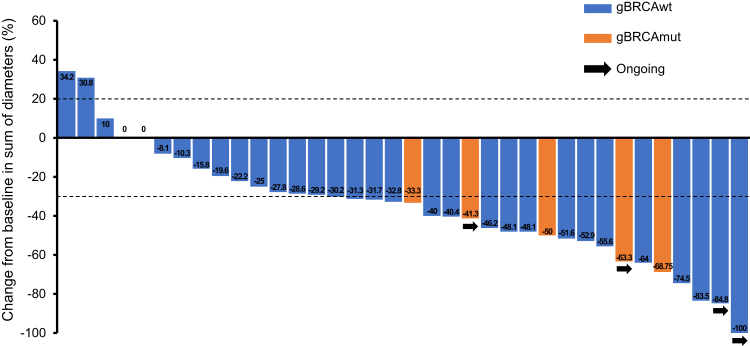
Change in the sum of the longest diameters of target lesions at the time of best response as per RECIST v1.1 (efficacy-evaluable population). Each bar represents one patient with evaluable tumour outcomes.

**Fig. 3:**
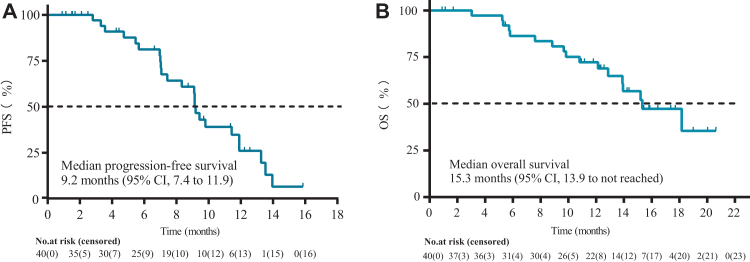
Kaplan–Meier curves for (A) progression-free survival (PFS) and (B) overall survival (OS) (intention-to-treat population).

By data cut-off, the ORR in the ITT population was 50.0% (95% CI 33.8–66.2), including one complete response and 19 partial responses (Table 2). Four patients withdrew without post-baseline tumour assessments, leaving 36 patients comprising the efficacy-evaluable population, for which the ORR was 55.6% (95% CI 38.1–72.1). Upon best overall response, 31 (86%) of the 36 efficacy-evaluable patients had a decrease in the sum of diameters of target lesions (Fig. 2). For patients with baseline g*BRCA*mut, the ORR was 100% (95% CI 47.8–100, 5/5 patients), while that for their g*BRCA* wildtype counterparts was 42.9% (95% CI 26.3–60.6; 15/35 patients). The ORR was 50.0% for the 30 patients with high-grade serous adenocarcinoma who underwent tumour evaluation. Post-hoc subgroup analysis did not identify other factors that affected the PFS (Supplementary Fig. S1). The median TTR, DOR, and PFI were 2.8 months (95% CI 1.6–4.9), 6.9 months (95% CI 4.2–9.7), and 13.2 months (95% CI 11.0–17.7), respectively (Supplementary Figs. S2–S4). With 24 PFS events (all were disease progression), the median PFS was 9.2 months (95% CI 7.4–11.9), and the 6-month PFS rate was 81.17% (95% CI 62.75–91.08) (Fig. 3A). There were 17 deaths by data cut-off, and the median OS was 15.3 months (95% CI 13.9–not evaluable) (Fig. 3B). Prior use of antiangiogenics did not affect the PFS (Supplementary Fig. S5). Prior use of antiangiogenics and lines of chemotherapy did not affect the OS (Supplementary Figs. S6–S7).

**Table 2:** Tumour response assessment (N = 40).

Response	Patients
Objective response rate, % (95% CI)	50.0 (33.8–66.2)
Best overall response	
Complete response	1 (3)
Partial response	19 (48)
Stable disease	14 (35)
Progressive disease	2 (5)
Not evaluable^a^	4 (10)
Disease control in efficacy-evaluable population^b^, % (95% CI)	97.2 (85.5–99.9)

Note: Data are presented as No. (%) unless indicated otherwise.

a The four patients withdrew from the study before undergoing the first post-baseline tumour assessment.

b Efficacy-evaluable population consisted of the 36 patients with results available for at least one post-baseline tumour assessment.

### Safety

The most common TEAEs (Table 3) of any grade were hypertension (22 patients, 55%), leukopenia (18 patients, 45%), and hand-foot syndrome (17 patients, 43%). Drug-related, grade ≥3 TEAEs were reported in 26 patients (68%), with the following reported in >1 patient: neutrophil count decrease (7 patients, 18%), hand-foot syndrome (6 patients, 15%), anaemia and hypertension (5 patients each, 13%), platelet count decrease and white blood cell count decrease (4 patients each, 10%), and hypertriglyceridemia and intestinal obstruction (3 patients each, 8%). All TEAEs documented are presented in Supplementary Table S2. No treatment-related death or myelodysplastic syndrome was recorded.

**Table 3:** Treatment-emergent adverse events reported in ≥20% of all treated patients (N = 40).

Treatment-emergent adverse events	Any grade No. (%)	Grade 1/2 No. (%)	Grade 3/4 No. (%)
Hypertension	22 (55)	17 (43)	5 (13)
Leukopenia	18 (45)	14 (35)	4 (10)
Hand-foot syndrome	17 (43)	11 (28)	6 (15)
Thrombocytopenia	15 (38)	11 (28)	4 (10)
Neutropenia	14 (35)	7 (18)	7 (18)
Hypertriglyceridemia	12 (30)	9 (23)	3 (8)
Heart rate increased	12 (30)	12 (30)	0
Pain	12 (30)	12 (30)	0
Haemoglobin reduction	10 (25)	5 (13)	5 (13)
Proteinuria	10 (25)	9 (23)	1 (3)
Poor appetite	8 (20)	7 (18)	1 (3)
Blood creatinine increased	8 (20)	8 (20)	0
Blood uric acid increased	8 (20)	8 (20)	0

TEAE-driven dose reductions occurred for anlotinib and niraparib in 21 patients and 19 patients, respectively, including 11 patients with dose reductions for both drugs. TEAE-driven dose interruptions occurred for anlotinib and niraparib in 14 patients and 21 patients, respectively, including 6 patients who dose-interrupted both drugs. Among the four patients who discontinued treatment due to TEAEs, two discontinued both drugs while one patient each discontinued niraparib or anlotinib only.

### Exploratory analysis

For the exploratory analysis of circulating tumour markers, 56 plasma samples were available from 12 patients. Three patients had baseline *BRCA* mutations in ctDNA and all three achieved objective responses, while only one of the eight patients with baseline wildtype *BRCA* achieved objective response (p = 0.024). Greater tumour size reductions at best response were observed in patients with baseline *SPEN* mutations compared to those without (p = 0.048; Supplementary Fig. S8A). Conversely, patients with baseline wildtype *PPM1D* experienced greater best-response tumour size reductions than those with *PPM1D* mutations (p = 0.048; Supplementary Fig. S8B). Higher variant allele frequencies of *PPM1D* were observed with increasing cycles of treatment (Supplementary Fig. S8C) and tumour change ratio (Supplementary Fig. S8D).

## Discussion

This study showed that niraparib–anlotinib combination therapy was active in PROC, providing clinical efficacy in terms of tumour response and PFS. The combination was well tolerated, and no new safety signal was observed compared to the individual single-agent safety profiles. Hence, the niraparib–anlotinib combination may provide a potential novel treatment regimen for patients with PROC, who currently have very limited treatment options.

Previous studies have confirmed the value of combining antiangiogenics and PARP inhibitors in treating platinum-sensitive ovarian cancer.^12^^,^^13^ In platinum-resistant diseases, single-agent PARP inhibitors have demonstrated ORRs of approximately 25%–35% and <5% in patients with and without *BRCA* mutations, respectively.^19^^,^^26^^,^^27^ Similarly, single-agent antiangiogenic TKIs yielded ORRs of 0%–20% in platinum-resistant diseases.^28^, ^29^, ^30^, ^31^, ^32^ In our study, 50% of the ITT population achieved an objective response, supporting the notion of augmented antitumour activity when combining the two drug classes. Since bevacizumab is widely recommended for advanced or recurrent ovarian cancer, most patients in this study had received prior antiangiogenic treatments, and our results suggest that a treatment history with antiangiogenics does not seem to affect the efficacy of subsequent application of anlotinib in combination with niraparib.

Another angiogenic–PARP inhibitor combination that has been evaluated in PROC is cediranib plus olaparib. From four phase 2 studies (NCT02345265, CONCERTO, BAROCCO, OCTOVA), it has been observed that cediranib–olaparib could achieve ORRs of 15%–20% in patients with or without g*BRCA*mut^16^^,^^17^^,^^33^; the median PFS of 5.1–5.6 months were numerically longer than that with weekly paclitaxel (3.1–3.9 months), but statistical superiority was not established.^15^^,^^16^ In our study, the ORR of 50% was achieved in a patient group where only 13% (5/40) of patients had g*BRCA*mut. This is encouraging and suggests that niraparib–anlotinib may overcome the biomarker restrictions of PARP inhibitor monotherapies and benefit the wider population of platinum-resistant patients. Also worth noting is that the median PFS in our study was more than half a year, translating to considerably prolonged PFIs. Some of our patients showed responsiveness to further platinum-containing chemotherapy after the end of study treatment, an intriguing observation that requires further research to verify.

Molecular biomarkers associated with significant efficacy could not be identified in our exploratory analysis of ctDNA. In our analysis, the presence and accumulation of *PPM1D* mutations appeared to correlate with poorer tumour responses. Previous studies suggest that somatic mosaic mutations in *PPM1D* may indicate heavier prior exposure to selective pressure for cells with an impaired DNA damage response.^34^ *PPM1D* is implicated in regulating the chemosensitivity of OVCA cells,^35^ and in diffuse intrinsic pontine glioma (DIPG), PPM1D inhibition was found to sensitize *PPM1D*-mutant DIPG cells to PARP inhibitor treatment.^36^ Our result is consistent with these earlier findings, which suggest that *PPM1D* mutations may be related to PARP inhibitor resistance and thus to poorer tumour responses. Our analysis also showed better tumour responses for patients with *SPEN* mutations. Currently, there are very limited reports on *SPEN* and ovarian cancer. The relationship between *SPEN* and tumour responses and the underlying mechanisms await further investigation.

Combination therapies should offer enhanced efficacy without incremental toxicity. In our study, no new safety signals were observed with niraparib–anlotinib compared with the respective single-agent safety profiles.^37^^,^^38^ Haematological toxicity (especially anaemia) occurred less frequently than in other niraparib trials.^37^ This may be related to the hypoxic environment created by anlotinib, which promotes erythropoiesis through hypoxia-inducible factor-regulated expression of erythropoietin.^39^ Unlike earlier studies of niraparib using the fixed starting dose of 300 mg,^20^ our study used bodyweight-directed individualized starting doses, whereby most patients started niraparib at 200 mg. Additionally, with rigorous routine blood tests, study drugs were immediately discontinued once the patient's platelet level decreased to below 100 000/μL. These measures likely contributed to the lower incidence of grade 3–4 thrombocytopenia observed. The incidence of common adverse events associated with anlotinib, such as hypertension, hand-foot syndrome, and certain biochemical abnormalities were not increased compared with previous studies.^38^ Regarding the protocol amendment (see *Procedures*) of starting dose reduction for anlotinib, although a relatively higher incidence of TEAEs (mainly hand-foot syndrome and hypertension) was noted for the first 23 patients enrolled who initiated anlotinib at 12 mg, the TEAEs were mostly of grade 1–2 and could be managed through protocol-specified dose modification. The protocol amendment was implemented to further optimize patient experience. Grade 3–4 TEAEs in this study were mainly neutropenia, hand-foot syndrome, hypertension, and anaemia, which could be managed and controlled. Notably, late-line patients tend to have poor bone marrow function and are thus more prone to grade ≥3 TEAEs related to bone marrow suppression. We plan to further explore the prevention and management of TEAEs through possible dose optimization in future studies.

Most small-molecule TKIs such as anlotinib, cediranib, pazopanib, and apatinib are metabolized via the CYP450 pathway, as are most PARP inhibitors such as olaparib, rucaparib, pamiparib, and fluzoparib. Consequently, the maximal tolerated doses of antiangiogenics and PARP inhibitors in combination are often lower than the full clinical doses of the single agents. In the studies of cediranib–olaparib, either cediranib (at 20 mg QD instead of 30 mg QD) or olaparib (at 200 mg twice a day [BID] instead of 300 mg BID) needed to be used at a reduced dose level; further dose reduction through intermittent dosing improved the safety but at the expense of PFS.^15^^,^^33^^,^^40^ In contrast, niraparib is metabolized by carboxylesterases and has minimal drug-to-drug interactions with agents metabolized via the CYP450 pathway.^18^ In our study, niraparib and anlotinib were both administrated at the usual clinical doses and were well tolerated. The possibility of using the full doses is conducive for optimizing efficacy, and also allows more room for dose adjustments when necessary to manage TEAEs and improve patients’ quality of life.

This study had several limitations. Firstly, it was a single-arm, phase 2 study with a small sample size. Although we observed an increased ORR and a trend of prolonged PFS, the long-term efficacy will need to be validated in a large trial. Secondly, the relationship between HRD status and efficacy could not be determined due to difficulties in obtaining valid specimens from the heavily pre-treated patient population. Thirdly, this study excluded patients with prior exposure to PARP inhibitors, who would likely constitute considerable proportions of PROC patients given the widespread front-line application of PARP inhibitors in ovarian cancer. We have planned an expansion study (ANNIE Plus) to include the patients with prior PARP inhibitor treatment to strengthen the clinical relevance of our findings. Fourthly, the COVID-19 pandemic precluded regular on-site follow-ups for some patients. We performed close telephone follow-ups, delivered study medications by post, and guided the patients to local hospitals where possible to minimize the impact of reduced on-site follow-ups. ANNIE was an early-phase, non-randomised study intended for initial exploration of niraparib–anlotinib in PROC, while the clinical application of this combination awaits support by further evidence, such as that to be generated from randomised controlled trials. Results of the ANNIE and ANNIE Plus studies would hopefully inform future such randomised controlled trials.

In conclusion, the niraparib–anlotinib combination showed promising antitumour activity and tolerable toxicity in patients with PROC. This oral, chemo-free combination may represent a potential new treatment option for patients with PROC.

## Contributors

GCL, YLF, and JL contributed equally to this work. Conception and design: JHL, YLF, GCL; administrative support: JHL, AL, JJ, BHK; provision of study materials or patients: JHL, AL, JJ, BHK, MZ, YX, JDL, YWH, CYZ, HH, TW, QDH; collection and assembly of data: GCL, JL, TD, AJY, LY; data analysis and interpretation: GCL, JL, LY; manuscript writing: All authors; final approval of manuscript: all authors; accountable for all aspects of the work: all authors. All authors were responsible for the decision to submit the report for publication. All authors had unrestricted access to all the study data, have read and approved the version of the article to be submitted, and accept responsibility for all content and editorial decisions. GCL and JHL had directly accessed and verified the underlying data.

## Data sharing statement

The authenticity of this article has been validated by uploading the key raw data onto the Research Data Deposit public platform (www.researchdata.org.cn), with the approval RDD number RDDA2022410532. Further inquiries can be directed to the corresponding author. Deidentified individual participant data will be made available to researchers who provide a methodologically sound proposal, subject to approval by the ethics committee at Sun Yat-Sen University Cancer Center, to achieve aims in the approved proposal. Proposals should be directed to the corresponding author. The study protocol is available in the appendix.

## Declaration of interests

All authors declare no competing interests.

## Appendix A Supplementary data

Supplementary Tables S1 and S2 and Figs. S1–S8

Captions for Supplementary Materials

ANNIE Study Protocol
