# Supplementary material for: A novel combination of niraparib and anlotinib in platinum-resistant ovarian cancer: Efficacy and safety results from the phase II, multi-center ANNIE study
Source: eClinicalMedicine. 2022 Nov 30;54:101767. doi: 10.1016/j.eclinm.2022.101767 (PMC9793276; doi:10.1016/j.eclinm.2022.101767)
Supplement: Supplementary Tables S1 and S2 and Figs. S1–S8 [file mmc1.pdf]

Supplementary Materials

Table of Content

Supplementary Table 1. Prior Antiangiogenic Treatments Received by Patients from the ANNIE Study (N=40) 2

Supplementary Table 2. Treatment-Emergent Adverse Events Documented in ANNIE (n=40) 3

Supplementary Figure 1. Post-hoc subgroup analyses of hazard ratios for progression-free survival (PFS)....................5

Supplementary Figure 2. Kaplan-Meier curve for time to response (TTR) for the 20 patients who achieved an objective response....................6

Supplementary Figure 3. Kaplan-Meier curve for duration of response (DOR) for the 20 patients who achieved an objective response....................7

Supplementary Figure 4. Kaplan-Meier curve for platinum-free interval (PFI)....................8

Supplementary Figure 5. Kaplan-Meier curves for progression-free survival (PFS) in subgroups with and without prior antiangiogenic treatment....................9

Supplementary Figure 6. Kaplan-Meier curves for overall survival (OS) in subgroups with different numbers of prior lines of chemotherapy (post-hoc)....................10

Supplementary Figure 7. Kaplan-Meier curves for overall survival (OS) in subgroups with and without prior antiangiogenic treatment (post-hoc) 11

Supplementary Figure 8. Exploratory analysis of the relationship between circulating tumour markers and prognosis. 12

Supplementary Table 1. Prior Antiangiogenic Treatments Received by Patients from the ANNIE Study (N=40)

Prior antiangiogenic treatment	No. (%)
Received prior antiangiogenic treatment	
Yes	27 (68)
No	13 (33)
Prior antiangiogenic agents[a]	
Anlotinib	2 (5)
Apatinib	6 (20)
Bevacizumab	21 (53)
Thalidomide	1 (3)

[a]Three patients received multiple antiangiogenic agents prior to the ANNIE trial.

Supplementary Table 2. Treatment-Emergent Adverse Events Documented in ANNIE (n=40)

Treatment-emergent adverse events	No. (%)
Hypertension	22 (55)
Leukopenia	18 (45)
Hand-foot syndrome	17 (43)
Thrombocytopenia	15 (38)
Neutropenia	14 (35)
Pain	12 (30)
Heart rate increased	12 (30)
Hypertriglyceridemia	12 (30)
Proteinuria	10 (25)
Hemoglobin reduction	10 (25)
Poor appetite	8 (20)
Blood creatinine increased	8 (20)
Blood uric acid increased	8 (20)
Constipation	7 (18)
Nausea	7 (18)
Hoarseness	7 (18)
Asthenia	6 (15)
Abdominal distension	6 (15)
Hemorrhage	6 (15)
Lower urinary tract infection	6 (15)
Transaminases increased	6 (15)
Rash	5 (13)
Blood cholesterol increased	5 (13)
Ileus	5 (13)
Insomnia	4 (10)
Gingival pain	3 (8)
Oral ulcer	3 (8)
Weight loss	3 (8)
Dizziness	3 (8)
Chest tightness	2 (5)
Hypoproteinemia	2 (5)

Urinary occult blood	2 (5)
Hyperthyroidism	2 (5)
Hyperglycemia	2 (5)
Vomiting	2 (5)
Diarrhea	1 (3)
Perianal dermatitis	1 (3)
Blood thyrotropin increased	1 (3)
Intestinal perforation[a]	1 (3)

[a]One patient experienced intestinal perforation 15 days after enrolment and underwent emergency Hartmann surgery. Considering the intraoperative finding and pathological results, the investigator determined that the perforation was caused by tumor infiltration to the entire intestinal wall and tumor necrosis after treatment.

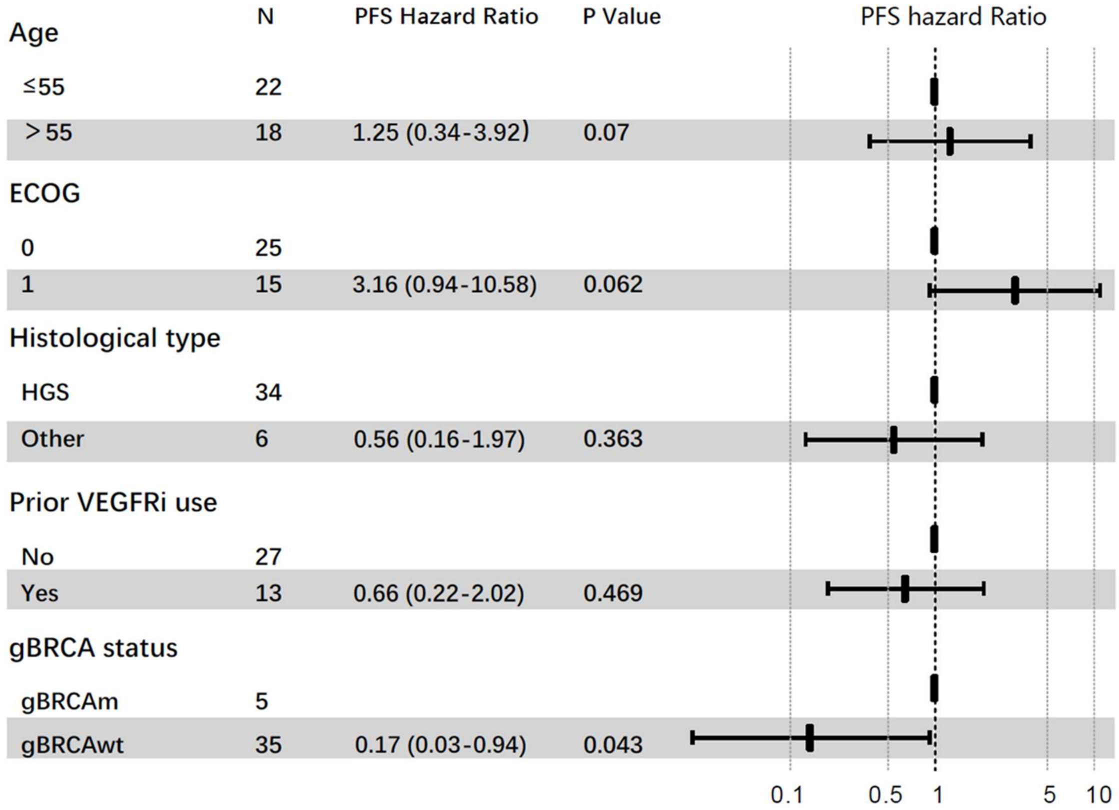

Supplementary Figure 1. Post-hoc subgroup analyses of hazard ratios for progression-free survival (PFS). ECOG, Eastern Cooperative Oncology Group; gBRCAm, germline BRCA mutation; gBRCAwt, germline BRCA wild type; HGS, high-grade serous carcinoma; HR, hazard ratio; VEGFRi, vascular endothelial growth factor receptor inhibitor.

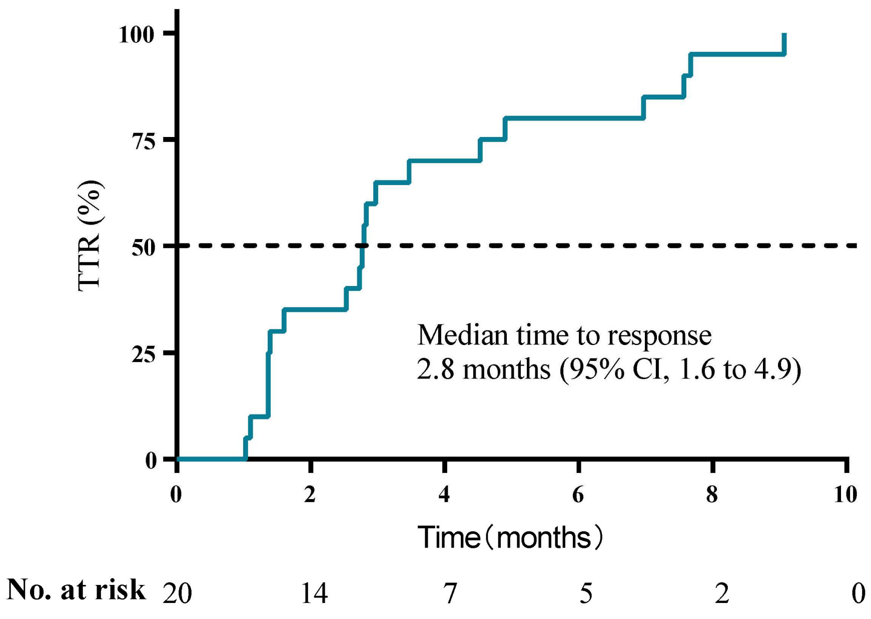

Supplementary Figure 2. Kaplan-Meier curve for time to response (TTR) for the 20 patients who achieved an objective response.

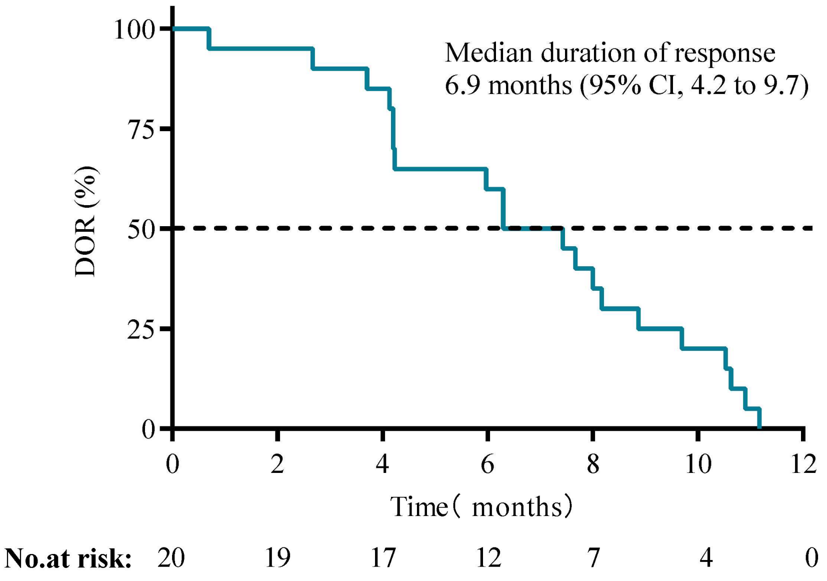

Supplementary Figure 3. Kaplan-Meier curve for duration of response (DOR) for the 20 patients who achieved an objective response.

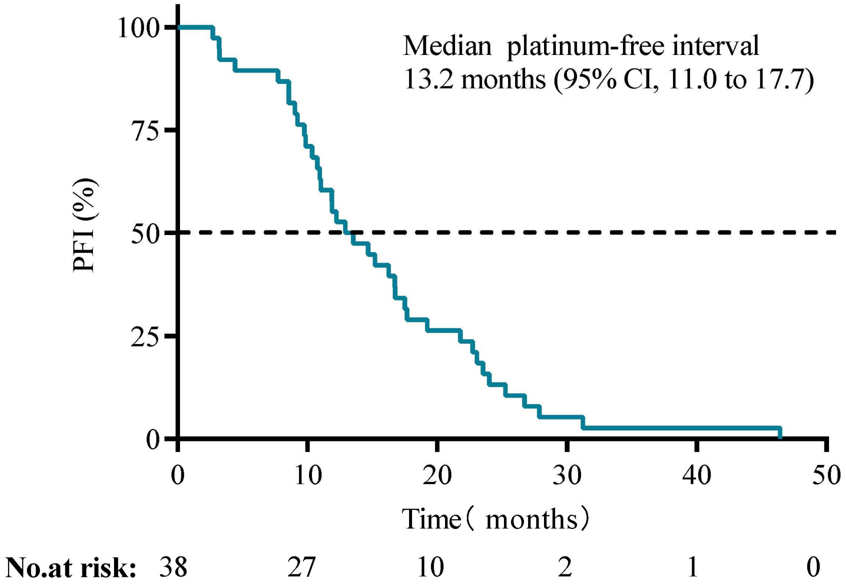

Supplementary Figure 4. Kaplan-Meier curve for platinum-free interval (PFI). In the intention-to-treat population (n=40), the exact time of the last round of platinum-containing chemotherapy could not be ascertained for two patients, who were thus excluded from this analysis.

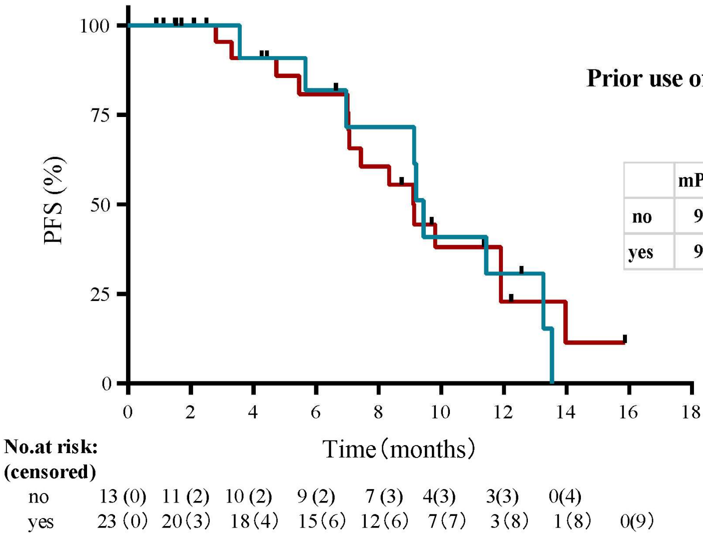

	mPFS	HR (95% CI)
no	9.4	1.07 (0.46 to 2.45)
yes	9.1	

Supplementary Figure 5. Kaplan-Meier curves for progression-free survival (PFS) in subgroups with and without prior antiangiogenic treatment. HR, hazard ratio; mPFS, median progression-free survival (months).

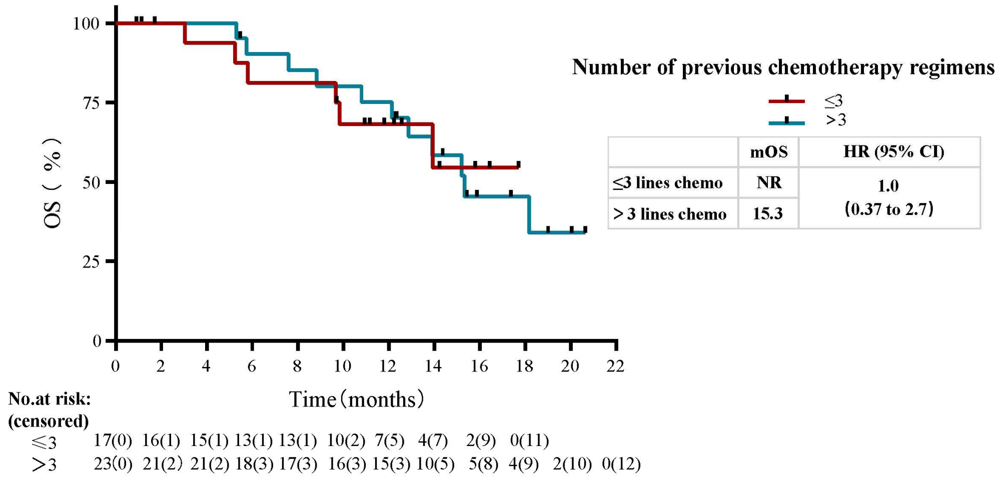

Supplementary Figure 6. Kaplan-Meier curves for overall survival (OS) in subgroups with different numbers of prior lines of chemotherapy (post-hoc). HR, hazard ratio; mOS, median overall survival (months); NR, not reached.

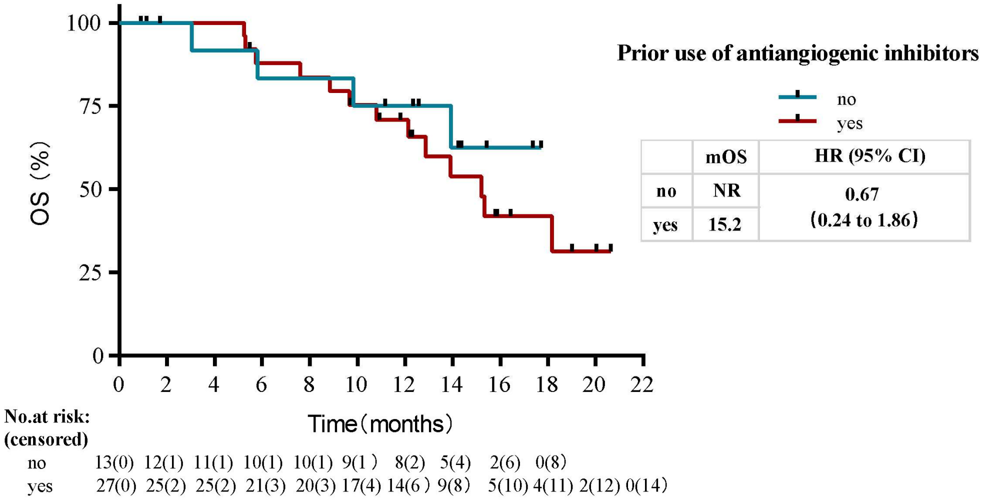

Supplementary Figure 7. Kaplan-Meier curves for overall survival (OS) in subgroups with and without prior antiangiogenic treatment (post-hoc). HR, hazard ratio; mOS, median overall survival (months); NR, not reached.

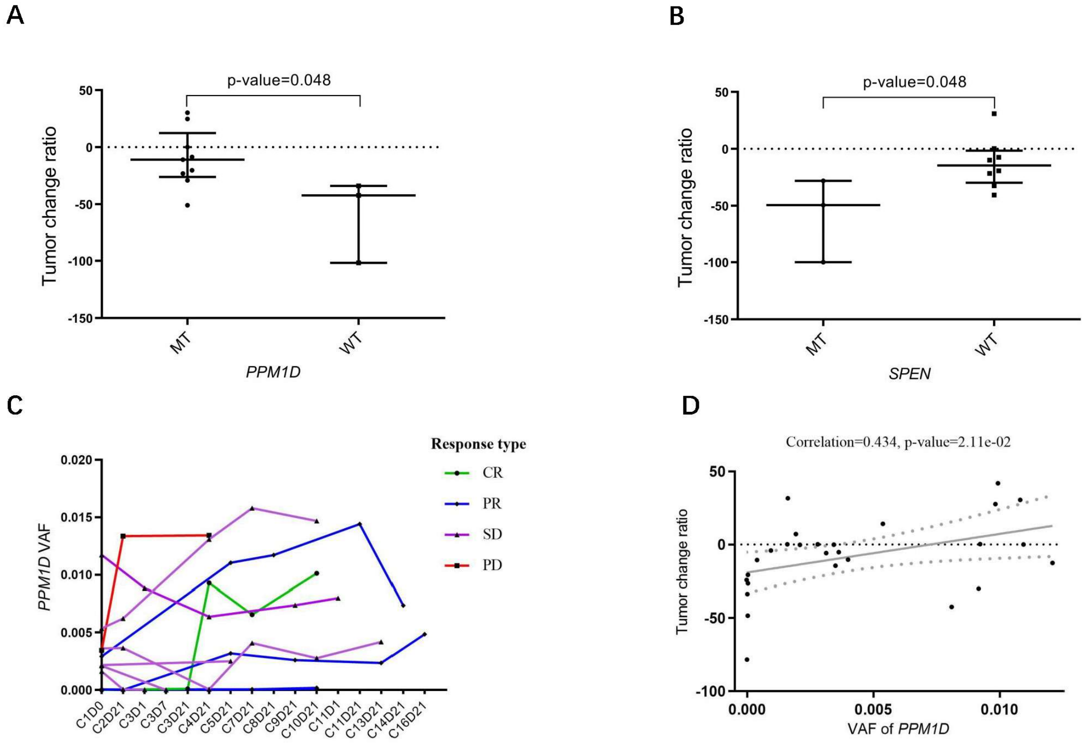

Supplementary Figure 8. Exploratory analysis of the relationship between circulating tumour markers and prognosis. (A–B) Greater reductions in tumour size at best response were observed with baseline mutated *SPEN* and wildtype *PPM1D*. (C) The VAF of *PPM1D* tended to increase with increasing cycles of treatment. (D) Increase in *PPM1D* VAF was correlated with increase in tumour size. C1D0, treatment cycle 1 day 0; CR, complete response; MT, mutant; PD, progressive disease; PR, partial response; SD, stable disease; VAF, variant allele frequency; WT, wild type.

Manuscript Appendix: ANNIE Study Protocol (Version 1.4)

A phase II clinical study of niraparib in combination with anlotinib in patients with platinum-resistant recurrent ovarian cancer

Protocol amendment version history:

Protocol	Version	Date	Amendment
Original	1.1	2020-1-14	N/A
1st amendment	1.2	2020-6-3	Provided additional recruitment materials and defined new routes of recruitment, to disseminate study information to more patients in need and expedite the progress of recruitment
2nd amendment	1.3	2020-7-27	In response to suggestion by lead investigator and in accordance with the Consensus on the Clinical Diagnosis, Treatment, and Prevention of Cancer- and Chemotherapy-Related Anemia in China (2019 edition), changed the inclusion criteria of "hemoglobin ≥10 g/dL" to "hemoglobin ≥9 g/dL" for better relevance to local clinical practice
3rd amendment	1.4	2020-10-8	Changed the starting dose of anlotinib to 10 mg

A phase II clinical study of niraparib in combination with anlotinib in patients with platinum-resistant recurrent ovarian cancer

**Study Title:**	An open-label, single arm, phase II trial of niraparib in combination with anlotinib in patients with platinum-resistant recurrent ovarian cancer, fallopian tube cancer, and primary peritoneal cancer (ovarian cancer)
**Project No.:**	CH1903005
**Version No. and Version Date:**	08Oct2020/Version 1.4
**Investigational Medicinal Product (IMP):**	Niraparib and anlotinib
**Study Phase:**	Phase II
**Sponsor:**	Sun Yat-sen University Cancer Center
**Signatory of Sponsor:**	Professor Jihong Liu
**Principal Investigator:**	Professor Jihong Liu
	Sun Yat-sen University Cancer Center
**Sponsors:**	Zai Lab (Shanghai) Co., Ltd., Chia Tai Tianqing Pharmaceutical Group Co., Ltd.
**Number of Cases Observed in The Trial:**	40 subjects

The document is accessible to investigators, research consultants or relevant personnel, Institutional Review Board/Independent Ethics Committee only. Disclosure of this document to third parties is prohibited.

Signature Page of the Investigator

Investigator's Statement

Protocol Title: An open-label, single-arm, phase II trial to evaluate the safety and efficacy of niraparib in combination with anlotinib in patients with platinum-resistant recurrent ovarian cancer, fallopian tube cancer, and primary peritoneal cancer (ovarian cancer)

Clinical Study No.: CH1903005

I have read the full text of the protocol, including all appendices.

By signing this agreement, I agree to conduct the clinical study in accordance with the study protocol, the International Conference on Harmonization (ICH), GCP guidelines, and applicable regulatory requirements after obtaining the approval of the Independent Ethics Committee (IEC)/ Institutional Review Board (IRB).

I will ensure that, under my direction, all personnel participating in the study will be informed of the contents of this study protocol and will receive all necessary guidance to perform the trial protocol.

Investigator

Jihong Liu MD pHD Director of GYO SYSUCC ______________ ______________

Contents

Signature Page of the Investigator 16

List of Abbreviations and Definitions of Terms 21

1 Introduction 23

1. 1.1 .... Ovarian Cancer 23
2. 1.2Background of PARP and PARP Inhibitors 23
3. 1.3Background of Homologous Recombination Deficiency (HRD) 24
4. 1.4Background of Niraparib 25

1.4.1 Phase I clinical trials 25

1.4.2 Phase II clinical trial (PR-30-5020-C) QUADRA[13] 26

1.4.3 Phase III clinical trial (PR-30-5011-C) NOVA10 27

5. 1.5Background of application of anti-angiogenic agents in ovarian cancer field 30
6. 1.6Background of Anlotinib 32
7. 1.7Theoretical Rationale for the Study 34

2 Study Design and Study Plan 34

8. 2.1 .... Overview 34
9. 2.2 .... Primary Objective 35
10. 2.3 .... Secondary Objectives 35
11. 2.4 .... Inclusion Criteria 35
12. 2.5 .... Exclusion Criteria 37
13. 2.6Patient Withdrawal and Replacement 39

2.6.1 End of treatment 39

2.6.2 Withdraw from the study ... 39

3 Study Management ... 40

14. 3.1 ... Study Restrictions ... 40

3.1.1 Granulocyte colony-stimulating factor (GCSF) ... 40

3.1.2 P-glycoprotein substrates ... 40

3.1.3 Other anti-cancer therapy ... 40

3.1.4 Vaccine ... 40

3.1.5 Contraception ... 41

3.1.6 Blood donation ... 42

15. 3.2Schedule of Activities ... 42

16. 3.3Schedule of Visits ... 42

3.3.1 Screening process/enrollment (Visit 1, Day -28 to Day -1) ... 42

3.3.2 During the study ... 43

3.3.3 Withdrawal from treatment for any reason (Visit is completed within 7 days after the last dose of study drug) ... 43

3.3.4 Continuous assessment ... 44

3.3.5 Unscheduled assessments ... 44

4 IMPs ... 45

17. 4.1 ... Anlotinib HRD ... 45

4.1.1 Management ... 45

4.1.2 Packaging, labeling and storage ... 45

4.1.3 Handling and destruction ... 45

18. 4.2 ... Niraparib 46

4.2.1 Characteristics ... 46

4.2.2 Management ... 46

4.2.3 Potential risk of niraparib ... 46

4.2.4 Packaging and labeling ... 46

4.2.5 Handling and destruction ... 46

4.2.6 Prior and concomitant medications ... 46

19. 4.3Dose modification of study drugs ... 48

5 Hypotheses and Statistical Method 54
20. 5.1 ..... Safety Analysis 55
21. 5.2Missing Data n/a 55
22. 5.3Interim Analysis n/a 55

6 Ethical, Legal and Administrative Provisions 55
23. 6.1 ..... GCP 55
24. 6.2 ..... Informed Consent 55
25. 6.3Approval and Amendment of Protocol 56
26. 6.4Confidentiality and Data Protection of Subjects 56
27. 6.5Source Data Verification 56
28. 6.6Quality Assurance, Review and Approval 56
29. 6.7 ..... Monitoring 56
30. 6.8Safety Monitoring and Reporting 57
6.8.1 Definition of AEs 57
6.8.2 Definition of SAEs 57
6.8.3 AE recording 57
6.8.4 AE assessment 58
6.8.5 Reporting of SAEs 58
6.8.6 Follow-up of AEs 59
6.8.7 Suspected unexpected serious adverse reactions (SUSARs) 59
6.8.8 Pregnancy 59
31. 6.9 ..... IMPs 59
6.9.1 Dispensing 60
6.9.2 Drug liability 60
32. 6.10 ..... Paper

publication 60
7 Schedule of Activities 61
8 Reference 63
Appendix 65
33. Appendix 1-Eastern Cooperative Oncology Group (ECOG) Performance Status Score 65

List of Abbreviations and Definitions of Terms

Abbreviations	English Terminology
ADP	Adenosine diphosphate
AE	Adverse event
ALT	Alanine aminotransferase
AML	Acute myeloid leukemia
AST	Aspartate aminotransferase
ATM	Ataxia-telangiectasia syndrome
AUC	Area under the plasma drug concentration-time curve
BER	Base excision repair
BRCA	Breast cancer susceptibility gene
CBC	Complete blood count
CFI	Chemotherapy-free interval
CI	Confidence interval
C_{max}	Maximum plasma concentration
$C_{ss\ max}$	Maximum plasma concentration at steady state
CR	Complete response
CRF	Case Report Form
CT	Computerized tomography
CTCAE	Common Terminology Criteria for Adverse Events
ECG	Electrocardiogram
ECOG	Eastern Cooperative Oncology Group
EMA	European Medicines Agency
ESMO	European Society for Medical Oncology
FDA	Food and Drug Administration
g*BRCA*mut	Germline *BRCA* mutation
GCP	Good Clinical Practice
HR	Hazard ratio
HRD	Homologous recombination deficiency
ICF	Informed Consent Form
ICH	International Conference on Harmonization
ITT	Intent-to-treat population
MPV	Mean platelet volume
MDS	Myelodysplastic syndrome
NHEJ	Non-homologous end joining
MedDRA	Medical Dictionary for Regulatory Activities
MRI	Magnetic Resonance Imaging
NCCN	National Comprehensive Cancer Network
OS	Overall survival
PARP	Poly (ADP-ribose) polymerase
PD	Progressive disease

Abbreviations	English Terminology
PFS	Progression-free survival
P-gp	P-glycoprotein
PR	Partial response
QD	Once daily
RECIST	Response Evaluation Criteria in Solid Tumors
SAE	Serious adverse event
SD	Stable disease
SUSAR	Suspected unexpected serious adverse reaction
VEGF	Vascular endothelial growth factor
TKI	Tyrosine kinase inhibitor

Introduction

Ovarian Cancer

Ovarian cancer is one of the most common malignancies of the female reproductive system, and is also one of the leading causes of death from gynaecological malignancy in China. In 2015, the number of new cases of ovarian cancer in China was ~52,100, whereas the number of deaths was ~22,500 [1].

Early-stage ovarian cancer is generally asymptomatic, approximately 75% of women present with advanced disease at diagnosis. The standard treatment for ovarian cancer mainly includes surgical resection (such as cytoreductive surgery) and combination chemotherapy consisting of paclitaxel and platinum. However, the vast majority of patients will experience disease recurrence 2 years after the above treatment. They gradually develop resistance to platinum-containing chemotherapy and become less tolerant to the toxicity of chemotherapeutic agents, with shorter progression-free survival (PFS), fewer subsequent effective treatments and worse prognosis. For ovarian cancer patients with distant metastasis, the five-year survival is only 29% [2].

At present, the treatment of recurrent ovarian cancer depends on the interval between the date of the last dose of the previous line of therapy and the date of relapse. Among them, platinum-resistant recurrent ovarian cancer is defined as tumor progression during or within six months after the last dose of platinum-based therapy [3,4]. According to the guidelines issued by the National Comprehensive Cancer Network (NCCN) and the European Society of Medical Oncology (ESMO), patients who are not considered suitable for platinum-based chemotherapy are treated with a platinum-free regimen such as weekly paclitaxel, liposomal doxorubicin and topotecan (alone or in combination with bevacizumab). However, platinum-free regimen has a lower response rate (10%-15%) and a shorter duration of response (3-4 months) in patients with platinum-resistant recurrent ovarian cancer. Besides, chemotherapeutic agents are inconvenient to be administrated, with high toxicity, which are difficulties in the treatment of patients with platinum-resistant recurrent ovarian cancer. In the study based on platinum-resistant recurrent ovarian cancer, there is an urgent need for a treatment mode with equal efficacy but less side effects. In addition, if there is a platinum-free treatment mode with the same efficacy after progressive disease (PD) (platinum may still be used in the later-line therapy when PD), it will prolong the platinum-free interval of patients, improve the quality of life and retain more later-line treatment options for these patients, which is also an unmet clinical demand at present.

Background of PARP and PARP Inhibitors

Poly (ADP-ribose) polymerase (PARP-1 and PARP-2) is a zinc-finger DNA binding protein used to detect DNA single-strand breaks and promote repair. After detecting DNA damage, PARP can transfer breaks to intracellular signaling and activate base-excision repair (BER) pathway[5], which is very important for cells with defects (homologous recombination deficiency) in high-fidelity

double-strand DNA repair mechanism. As described above, inactivation of some genes, e.g., *BRCA* 1/2 gene and ataxia telangiectasia mutated (*ATM*) gene, may lead to HRD. In the context of defects in DNA double-strand repair, cell DNA damage repair will rely more on the single-strand damage repair pathway; therefore, PARP inhibitors can have synergistic and enhanced effect of inducing cell death in ovarian cancer patients with widespread HRD due to germline BRCA mutations, etc., through blockade and inhibition of DNA single-strand damage repair in combination with DNA double-strand repair defects caused by HRD[6]. At the same time, the mechanism of inducing cell death of PARP inhibitors also suggests that it will also produce anti-cancer effect when applied to ovarian cancer patients with non-BRCA gene abnormality. Pre-clinical in vitro and in vivo studies have proven the efficacy of the target. Therefore, targeted therapy directed at PARP has a solid scientific foundation in patients with ovarian cancer.

In recent years, multiple clinical studies have showed the efficacy of PARP inhibitors to recurrent ovarian cancer [7-9]. Niraparib is a potent and highly selective PARP-1/2 inhibitor developed by TESARO in the world (except China). In a phase III, randomized, placebo-controlled international multicenter clinical trial that has been completed recently, niraparib, as a maintenance treatment, significantly prolonged PFS of patients with platinum-sensitive recurrent ovarian cancer. To date, this is the first completed phase III confirmatory study that proved the clinical efficacy of PARP inhibitors in patients with recurrent ovarian cancer; what's more, niraparib showed significant efficacy in ovarian cancer patients carrying germline *BRCA* mutation and those carrying non-germline *BRCA* mutation[10]. As strongly backed by supporting data as described above, niraparib was granted accelerated review and priority review by FDA in September and December 2016, respectively, and obtained accelerated approval for marketing 3 months in advance by FDA in March 2017 for the maintenance treatment of platinum-sensitive recurrent ovarian cancer, and was subsequently approved by the European Medicines Agency (EMA) in November 2017 for maintenance treatment of platinum-sensitive recurrent high-grade serous ovarian cancer. In the recently reported study (TOPACIO/KEYNOTE-162: niraparib + PD-1 monoclonal antibody in patients with platinum-resistant recurrent ovarian cancer), the combination of the two drugs has also achieved an objective response rate (ORR) of 18% and a disease control rate (DCR)of 60%, suggesting that niraparib also has good efficacy in platinum-resistant recurrent ovarian cancer. Zai Lab obtained the exclusive right of research & development, production and sales of niraparib in China (including Hong Kong and Macau) in September 2016, with number of ZL-2306.

Background of Homologous Recombination Deficiency (HRD)

Complete loss of function or inactivation of both *BRCA* alleles is often found in women with germline *BRCA* (*gBRCA*) mutations. The loss of *BRCA* function results in the inhibition of homologous recombination pathways of DNA repair, thus impairing DNA double-strand break repair. Therefore, these tumors often use alternative error-prone DNA repair pathways, such as non-homologous end joining (NHEJ). Therefore, the loss of homologous recombination in ovarian cells significantly contributes to the accumulation of mutations, eventually leading to the

occurrence of ovarian neoplasm [11]. Many DNA repair processes involve PARP-1 and PARP-2 zinc-finger DNA binding enzymes, which can detect DNA damage and convert it into intracellular signals to promote DNA repair. For example, PARP inhibitors can block DNA repair by inhibiting BER. In normal cells, homologous recombination repair pathway can compensate for PARP-mediated BER inhibition, so as to maintain the fidelity of DNA repair. In cells with homologous recombination repair defects, e.g., cells carrying *BRCA*1 and *BRCA*2 mutations, inhibition of PARP may lead to non-repairability and replication fork collapse of double strand break, and increase in the use of error-prone pathway NHEJ. The damages may result in instability of genome, finally leading to cell death. Complementarity between the cell deficiency and drug inductive effect is called synergistic lethal effect[12], by which PARP inhibitors can selectively kill cancer cells with homologous recombination deficiency and other DNA repair defects.

Background of Niraparib

Niraparib is an oral, potent, highly selective PARP-1 and PARP-2 inhibitor. It can co-crystallize with the catalytic domain of human PARP-1 and inhibit the activities of PARP-1 and PARP-2 in vitro, with a half-maximal inhibitory concentration (IC_{50}) of 3.8 and 2.1 nM, respectively. In the cultured cells, niraparib inhibits PARP-dependent PARylation caused by DNA damage, with IC_{50} and IC_{90} of 4 nM and 40 nM, respectively. Niraparib has demonstrated selective anti-proliferative activity in cancer cell lines that have been silenced for *BRCA*1/2 or carry *BRCA*1/2 mutations, which was 25-200 times higher than their wild type counterparts. Tumors in the *BRCA* and *ATM* models can regress after xenograft tumor-bearing mice are treated at clinically relevant doses. At these dose levels, 90% PARP inhibition for up to 24 hours can be observed in tumors after a single dose, which is stronger and more lasting than PARP inhibition in PBMC, and the inhibition level in PBMC is 50% or lower by 24 hours post-dose. In addition, niraparib has been evaluated in more than 30 models of ovarian cancer patient-derived xenograft (PDX) and cell line derived tumor xenograft, and tumor regression has been observed in *BRCA*1/2-mutant xenografts and *BRCA* wild-type HRD-positive models. Furthermore, tumor growth inhibition has also been observed in some HRD-negative models.

Phase I clinical trials

Five Phase I clinical studies (PN001, PN008, PN011, PN014, PR-30-5015-C) were conducted in 144 patients, as shown in Table 1. Patients participating in these studies received treatment when there are no significant safety concerns or PD evidence, and if the investigator considers such treatment to be in the best interest of the patient, the patient can continue study treatment, including the possibility of conducting the study with niraparib alone or in combination with other drugs.

Table 1: Phase I Clinical Trials of Niraparib

**Phase I**	
PN001	Phase I clinical study of niraparib in patients with advanced solid tumors or hematological malignancies (completed)
PN008	Phase I clinical study of niraparib in combination with carboplatin for the treatment of advanced solid tumors (terminated due to non-safety reasons)
PN011	Phase I clinical study of niraparib in combination with doxorubicin for the treatment of advanced solid tumors (terminated due to non-safety reasons)
PN014	Phase I clinical study of niraparib in combination with temozolomide for the treatment of advanced solid tumors (completed)
PR-30-5015-C	Phase I clinical study on absorption, metabolism, and excretion of niraparib in patients with advanced solid tumors (completed)

Phase II clinical trial (PR-30-5020-C) QUADRA[13]

Study PR-30-5020-C is a phase II clinical trial mainly in 463 patients with recurrent high-grade serous ovarian cancer who have received at least three-line therapy. This study is intended to evaluate the anti-cancer activity, tolerability and safety data of niraparib alone in enrolled subjects at this stage. At present, there are still 21 patients receiving treatment in the group, and the enrolled patients have previously received 4 lines of chemotherapy on average, with a median follow-up time of overall survival (OS) of 17.2 months (IQR 14.9-19.8). Thirteen of 47 evaluable patients (who have received three- to four-line chemotherapy, without previous use of PARP inhibitors and with positive HRD status) achieved response with an OS of 28%, a median PFS of 5.5 months (3.5-8.2), and a median duration of sustained response of 9.2 months (5.9 to not achieved).

Table 2 lists the response of all PARP inhibitor-naïve patients according to different BRCA status, other molecular markers and platinum sensitivity. Table 4 summarizes proportion of patients with 24-week clinical benefit according to biomarkers and platinum sensitivity status, and the ratio with previous PFS. Preliminary data from this clinical trial also reveal promising prospects of niraparib monotherapy for ovarian cancer [13].

Table 2 Response of niraparib monotherapy for recurrent ovarian cancer

	BRCA-mutated (n = 63)	HRD positive* (n = 189)	HRD negative or unknown (n = 230)
Sensitive to the latest platinum-based chemotherapy	7/18 (39%)	14/53 (26%)	2/52 (4%)
Resistant to or fail platinum-based chemotherapy	10/37 (27%)	12/120 (10%)	5/169 (3%)
It is unknown whether patients are sensitive to platinum-based chemotherapy	1/8 (13%)	3/16 (19%)	1/9 (11%)
All patients	18/63 (29%)	29/189 (15%)	8/230 (3%)

HRD positive = homologous recombination deficiency, *HRD positive patients include those with BRCA-mutated and non-BRCA-mutated tumors.

Table 3 Summary of proportion of patients with 24-week clinical benefit by biomarkers and platinum sensitivity status, and PFS ratio (relative to previous one)

	BRCA-mutated (n = 63)	HRD positive* (n = 189)	HRD negative or unknown (n = 230)
Sensitive to the latest platinum-based chemotherapy	10/18 (56%)	21/53 (40%)	10/52 (19%)
Resistant to or fail platinum-based chemotherapy	2/37 (32%)	24/120 (20%)	18/169 (11%)
It is unknown whether patients are sensitive to platinum-based chemotherapy	2/8 (25%)	5/16 (31%)	5/9 (56%)
All patients	24/63 (38%)	50/189 (26%)	33/230 (14%)
Patients with stable condition and PFS ratio (relative to previous one) > 1.3	9/25 (36%)	23/72 (32%)	39/103 (38%)
Patients with stable condition and PFS ratio (relative to previous one) > 1.0	11/25 (44%)	30/72 (42%)	47/103 (46%)

HRD positive = homologous recombination deficiency, *HRD positive patients include those with BRCA-mutated and non-BRCA-mutated tumors.

Phase III clinical trial (PR-30-5011-C) NOVA10

Study PR-30-5011-C is a double-blind, randomized (2:1), placebo-controlled study in histologically confirmed platinum-sensitive high-grade serous ovarian cancer patients with or without g*BRCA*ms and who have responded to their most recent chemotherapy containing a platinum agent. These patients were enrolled and grouped prospectively by comprehensive Myriad BRAC Analysis® results. The patient must have received at least 2 previous courses of platinum-containing chemotherapy, have responded to the last treatment, and have no measurable lesion of > 2 cm after the last treatment, with normal CA-125 (or decrease > 90%). The primary objective of this study is to evaluate efficacy of niraparib as maintenance therapy in patients who have platinum-sensitive recurrent high-grade serous ovarian cancer as assessed by the PFS. Stratified statistical analysis of PFS in non-g*BRCA*mut cohort was carried out. First, the HRD-positive tumor group was tested. If the first test result was statistically significant, then the overall non-g*BRCA*mut cohort was tested subsequently. There were 2 independent patient cohorts in this study: 1 cohort of g*BRCA*mut patients and 1 cohort of non-g*BRCA*mut patients. These patients received study treatment on Day 1 and every cycle (28 days) thereafter until discontinuation of study treatment. Three capsules of niraparib or placebo (strength of 100 mg) were orally administered once daily (QD) continuously. A total of 203 patients were enrolled in the g*BRCA*mut cohort and 350 patients were enrolled in the non-g*BRCA*mut cohort. Overall, 553 patients were randomized to niraparib (n=372) or placebo (n=181). Preliminary efficacy data (as of the data cut-off of 30May2016) of ITT population (n=553) and safety population (n=546) are available. Two hundred and sixty-six patients are still on treatment; and the enrollment has been closed for this study.

In all three prospectively defined main patient populations (g*BRCA*mut cohort, HRD-positive non-g*BRCA*mut cohort and overall non-g*BRCA*mut cohort) of this study, niraparib had the primary study endpoint (PFS) prolonged relative to placebo, as shown in Table 4.

Table 4: PFS for the primary efficacy cohorts (ITT population, N = 553)

Treatment	Median PFSa (95% CI) (month)	Hazard Ratio (95% CI), P value c	Percentage of patients without progression or death at following time points d:		
			6 months	12 months	18 months
**g*BRCA*mut** cohort					
Niraparib (n = 138)	21.0 (12.9, NE)	0.27 (0.173, 0.410) p < 0.0001	80%	62%	50%
Placebo (n=65)	5.5 (3.8, 7.2)		43%	16%	16%
**HRDpos** cohort					
Niraparib (n = 106)	12.9 (8.1, 15.9)	0.38 (0.243, 0.586) p < 0.0001	69%	51%	37%
Placebo (n=56)	3.8 (3.5, 5.7)		35%	13%	9%
Non-**g*BRCA*mut** cohort					
Niraparib (n = 234)	9.3 (7.2, 11.2)	0.45 (0.338, 0.607) p < 0.0001	61%	41%	30%
Placebo (n=116)	3.9 (3.7, 5.5)		36%	14%	12%

Source: PR-30-5011-C (NOVA main study) CSR

Abbreviations: *BRCA* = Breast cancer susceptibility gene; CI = Confidence interval; g*BRCA*mut = Germline *BRCA* mutation; HRDpos = Homologous recombination deficiency positive; ITT = Intent-to-treat; NE = Not estimated; non-g*BRCA*mut = non-germline *BRCA* mutation; PFS = Progression-free survival

a PFS is defined as the time from randomization to progression or death.

b Niraparib: placebo, based on the stratified Cox proportional hazard model using stratification factors of randomization.

c Based on stratified Log rank test using stratification factors of randomization.

d Product-limit estimate. Confidence intervals were constructed using logarithmic transformation.

Secondary endpoints, including chemotherapy-free interval (CFI), time to first subsequent therapy (TFST) and progression-free survival 2 (PFS2), confirmed that niraparib had PFS benefit in both cohorts[18], which proved that niraparib did not reduce the response to subsequent therapy, and also proved the continuous treatment effect of niraparib. There was no evidence that niraparib treatment had an adverse effect on OS.

No deaths were reported during the study. The majority of patients in both cohorts experienced at least 1 TEAE, including all 367 patients (100%) treated with niraparib and 171 patients (95.5%) treated with placebo (Table 5). Table 5 lists Grade 3/4 AEs occurring in ≥ 5% patients.

Table 5: AE Summary of PR-30-5011-C NOVA Main Study

AEs	Niraparib N = 367 n (%)	Placebo N = 179 n (%)
Any TEAEs	367 (100.0)	171 (95.5)
Nausea	270 (73.6)	63 (35.2)
Anaemia	178 (48.5)	12 (6.7)
Platelets decreased	169 (46.0)	6 (3.4)
Fatigue	168 (45.8)	58 (32.4)
Constipation	146 (39.8)	36 (20.1)
Vomiting	126 (34.3)	29 (16.2)
Headache	95 (25.9)	17 (9.5)
Decreased appetite	93 (25.3)	26 (14.5)
Insomnia	89 (24.3)	13 (7.3)
Abdominal pain	83 (22.6)	53 (29.6)
Platelet count decreased	74 (20.2)	4 (2.2)
Difficulty breathing	71 (19.3)	15 (8.4)
Hypertension	71 (19.3)	8 (4.5)
Diarrhea	70 (19.1)	37 (20.7)
Neutrophils reduced	66 (18.0)	6 (3.4)
Dizzy	61 (16.6)	13 (7.3)
Weakness	58 (15.8)	16 (8.9)
Cough	55 (15.0)	8 (4.5)
Back pain	49 (13.4)	21 (11.7)
Neutrophil count decreased	49 (13.4)	5 (2.8)
Arthralgia	43 (11.7)	22 (12.3)
Dyspepsia	42 (11.4)	17 (9.5)
Nasopharyngitis	41 (11.2)	13 (7.3)
Urinary tract infection	38 (10.4)	11 (6.1)
Palpitation	38 (10.4)	3 (1.7)
Taste disorder	37 (10.1)	7 (3.9)
Myalgia	30 (8.2)	18 (10.1)
Abdominal distention	28 (7.6)	22 (12.3)

Summary of hematologic AEs: At present, the common AEs of PARP inhibitors used clinically are hematologic AEs, including thrombocytopenia, neutropenia and anaemia. Hematologic AEs have also been summarized in our clinical study set.

A. Thrombocytopenia: overall, 186/384 (49%) patients had thrombocytopenia in the ovarian cancer study; of these, 28% had Grade 3 or higher thrombocytopenia and 8% had SAEs. Inter-study data analysis showed the highest incidence of all grades and Grade 3 or higher thrombocytopenia events in the QTc sub-study. The RADAR analysis after NOVA showed that the ratio of Grade 3 or higher thrombocytopenia events in

patients with body weight ≥ 77 kg and platelet count > 150,000/uL to those with body weight < 77 kg or platelet count < 150,000/ul was 39.3%: 16.1%, and the differentiated selection of starting dose was performed in both the subsequent PRIMA clinical trial and QUADRA clinical trial according to body weight and platelet count. In patients with body weight < 77 kg or platelet count <150,000/uL, niraparib 200 mg QD was selected as the starting dose, and the safety data was improved. Generally, thrombocytopenia could be managed clinically, and only a few patients discontinued the study drug due to the TEAE; a total of 12 patients (3%) in the NOVA study discontinued the study drug due to abnormal laboratory results, and similar incidences were observed in each study. According to the requirements of the protocol, dose interruption and reduction of study drug were required for patients with platelet count low. The study drug was interrupted in 133 patients (35%) and the dose was reduced in 62 patients (16%) due to the AE.

B. Anaemia: Including macrocytic anaemia, aregenerative anaemia, normochromic normocytic anaemia and haemoglobin decreased. Overall, 173/384 (45%) patients reported anaemia events in the ovarian cancer treatment study set; of these, 21% reported Grade 3 or higher anaemia and 3% reported SAEs. Generally, anaemia events could be managed clinically, and only a few patients discontinued the study drug due to the TEAE; a total of 5 patients (1%) discontinued the study drug due to abnormal laboratory results in the ovarian cancer treatment study set. According to the requirements of the protocol, dose interruption and reduction of study drug were required for patients with low haemoglobin. The study drug was interrupted in 50 patients (13%) and the dose was reduced in 20 patients (5%) due to the AE.

C. Neutropenia: Overall, 83/384 (22%) patients reported neutropenia events in the ovarian cancer treatment study set; of these, 14% reported Grade 3 or higher neutropenia and 2% reported SAEs. Review of various studies showed that the overall incidence of neutropenia event was higher in Study PN001 and Sub-study QTc, and the incidence of Grade 3 or higher events was the highest in Sub-study QTc. Generally, neutropenia could be managed clinically, and only a few patients discontinued the study drug due to the TEAE; a total of only 2 patients (< 1%) discontinued the study drug due to this abnormal laboratory result in the ovarian cancer treatment study set. According to the requirements of the protocol, dose interruption and reduction of study drug were required for patients with absolute neutrophil count (ANC) decreased. The study drug was interrupted in 35 patients (9%) and the dose was reduced in 13 patients (3%) due to the AE.

Background of application of anti-angiogenic agents in ovarian cancer field

Protein Tyrosine Kinase (PTK) signaling pathway is related to the proliferation, differentiation and migration of tumor cells. The interference or blocking of PTK pathway can be used to affect the growth of tumor cells [14]. Of these, vascular endothelial growth factor (VEGF) is the main

related mediator. VEGF promotes the proliferation and migration of vascular endothelial cells from abnormal tumors and increases the permeability of abnormal vessels by binding to VEGF receptor (VEGFR), while anti-angiogenic agents play a role in trimming and normalizing tumor neovascularization. Angiogenesis is an extremely complex process that requires the involvement of the vasculature, circulating endothelial cells, and pro-angiogenic mediators, including VEGF. Among them, VEGF/VEGFR is an important signaling pathway. VEGF activates intracellular signaling pathway by binding to VEGFR-l and VEGFR-2 expressed in tissue epithelial cells, leading to the proliferation of vascular endothelial cells and the neovascularization. The expression of VEGF is associated with the progression and stage of malignant tumors, ascites formation, shortened tumor-free survival, and poor overall prognosis. Immunohistochemical tests show that VEGF is expressed in tumors and metastatic tissues, and can be detected in malignant ascites and serum. The expressions of VEGF and VEGFR detected in tumor patients are independent prognostic factors.

Bevacizumab (Avastin) is a recombinant humanized immunoglobulin G1 (IgG1) monoclonal antibody, which can bind VEGF-A, inhibit its binding with VEGF receptor -2 (VEGFR-2), and then inhibit the biological effects of VEGF, including affecting vascular permeability, proliferation, migration and survival of endothelial cells, so as to achieve the effect of inhibiting tumor angiogenesis, growth and metastasis. In studies GOG213 and GOG218, the addition of bevacizumab did not bring the OS benefit, but the prolongation of PFS was statistically different from that in placebo group when compared to the traditional carboplatin plus paclitaxel regimen. Bevacizumab has been recommended by the US NCCN guidelines for the maintenance treatment of ovarian cancer [15].

Cediranib is an oral tyrosine kinase inhibitor (TKI) of VEGF receptors, which mainly inhibits VEGFR-1/2/3 and PDGFR. From 2011 to 2013, a phase II clinical trial was conducted in the United States to compare cediranib plus olaparib compared with olaparib alone in patients with platinum-sensitive recurrent ovarian cancer [16]. In this trial, 90 patients were included and randomized to receive cediranib plus olaparib or olaparib alone according to the ratio of 1:1. After an average follow-up of 46 months, the PFS (16.5 months) of the combination therapy group was significantly different from that (8.2 months) of the monotherapy group (HR 0.50 CI 0.3-0.83 P = 0.006). In the retrospective analysis based on BRCA factor, the PFSs of the combination therapy group and the monotherapy group in 47 BRCAm patients were 16.4 months and 16.5 months, respectively, and the HR was 0.76 (CI: 0.38-1.49, p=0.42), without significant difference; in 43 BRCAwt or unknown cases, the PFS of patients in the combination therapy group was significantly better than that in the monotherapy group (23.7 months vs 5.7 months HR 0.31 CI 0.15-0.66, P = 0.0013). In BRCA positive group (n=47), the ORR was 63% for olaparib alone (maintenance) versus 84% in the combination (P = 0.19), while in BRCAwt group, this figure was 32% versus 76% (P = 0.006), or 47.8%: 79.6% (P = 0.002) in the overall population.

According to the existing data, some anti-angiogenic agents for the maintenance treatment of ovarian cancer can benefit patients to different extents. Bevacizumab has been recommended by the NCCN guidelines for the maintenance treatment of ovarian cancer. Other new and not yet marketed TKI inhibitors such as Cediranib have also shown good efficacy when co-administered with PARP inhibitors in trials. Chemotherapeutic agents are inconvenient to be administrated, with high toxicity, which are difficulties in the treatment of patients with recurrent ovarian cancer. In the studies based on platinum-resistant recurrent ovarian cancer, a treatment mode that has side effects significantly reduced with equivalent efficacy is urgently needed. The early clinical trials of TKI inhibitors and PARP inhibitors have suggested that this combination may bring similar efficacy, smaller toxic and side effects, and give patients a longer chemotherapy-free interval, which is worthy of further exploration.

Background of Anlotinib

Anlotinib is a multi-targeted receptor tyrosine kinase inhibitor, which has significant inhibitory activity against angiogenesis related kinases, such as VEGFR1/2/3, FGFR1/2/3, and other tumor-related (e.g. cell proliferation) kinases such as PDGFRα/β, c-Kit, Ret, etc. It has broad spectrum of inhibitory action on tumor angiogenesis (such as Met, FGFR1/2/3). It can also target some kinases under study, such as Aurora-B, c-FMS, DDR1, and also has obvious inhibitory activity. It also has significant inhibitory activity against multiple kinase mutants, such as PDGFRα, cKit, Met, EGFR. The inhibitory activity against mutants is even more potent than that against wild-type counterparts.

Anlotinib hydrochloride capsule is a multi-target receptor tyrosine kinase inhibitor developed by Chia Tai Tianqing Pharmaceutical Group Co., Ltd., with independent intellectual property rights. In March 2011, it obtained the approval for clinical study from China Food and Drug Administration (SFDA: 2011L00661). Since May 2011, the phase I tolerability and pharmacokinetics study and the preliminary efficacy study of colorectal cancer have been completed in the Cancer Hospital, Chinese Academy of Medical Sciences. Meanwhile, multi-center phase II clinical studies of medullary thyroid cancer, soft tissue sarcoma, non-small cell lung cancer, and renal clear cell carcinoma, etc. have been conducted in China.

Due to the effective prolongation of OS and PFS by anlotinib for advanced NSCLC patients who have failed first-line and second-line treatment in phase III trial (ALTER0303) [17], anlotinib was formally approved in May 2018 for the treatment of advanced NSCLC patients who have failed first- and second-line treatment [18].

ALTER0303 study enrolled patients with locally advanced/metastatic non-small cell lung cancer who have progressed or are intolerant after receiving at least 2 kinds of systemic chemotherapy. EGFR-mutant or ALK-positive patients should have progressed or are intolerant after receiving corresponding targeted drug treatment. The ECOG score was 0 - 1 point; patients with bleeding tendencies, poorly controlled hypertension, abnormal coagulation function, and a 24-h urinary

protein quantitation of > 1.0 g were excluded from the trial. Two hundred and ninety-four patients were orally administered with anlotinib at a starting dose of 12 mg once daily (on for 2 weeks and off for 1 week), with a cycle of 3 weeks (21 days). 57.14% (168/294) subjects were treated for 6 cycles or more. Dose reduction (from 12 mg to 10 mg) occurred in 25 patients (8.50%) of the anlotinib group, and the second reduction (from 10 mg to 8 mg) occurred in 3 patients (1.02%). The main AEs leading to dose reduction were hand and foot skin reaction, hypertension, diarrhea, anorexia, stomatitis, function liver abnormal, proteinuria, lipids blood increased, and asthenia.

In the anlotinib group and the placebo group, the incidences of all grade AEs were 97.28% and 88.11%, respectively, and those of grade 3 and higher AEs were 47.28% and 18.18%, respectively. Table 6 presents adverse reactions with the incidence of ≥ 10% and Grade 3 or higher adverse reactions with the incidence of ≥ 2% (classified per NCI-CTC AE4.0) in the anlotinib and placebo groups in the ALTER0303 study.

Table 6 Summary of AEs of ALTER0303

AEs	Anlotinib (N = 294) n %		Placebo (N=143) n%	
	All grades	Grade ≥3	All grades	Grade ≥3
Asthenia	150(51.02)	1(0.34)	38(26.57)	0
Decreased appetite	133(45.24)	3(1.02)	43(30.07)	3(2.10)
Weight decreased	66(22.45)	0	12(8.39)	0
Pain	42(14.29)	2(0.68)	15(10.49)	2(1.40)
Diarrhea	103(35.03)	3(1.02)	21(14.69)	0
Oropharyngeal pain	83(28.23)	1(0.34)	10(6.99)	0
Stomatitis	68(23.13)	3(1.02)	4(2.80)	0
Vomiting	63(21.43)	1(0.34)	19(13.29)	0
Abdominal pain	53(18.03)	1(0.34)	13(9.09)	0
Nausea	52(17.69)	0	19(13.29)	0
Gingival pain	40(13.61)	0	2(1.40)	0
Cough	110(37.41)	2(0.68)	33(23.08)	1(0.70)
Difficulty breathing	90(30.61)	6(2.04)	32(22.38)	7(4.90)
Hoarseness of voice	66(22.45)	2(0.68)	7(4.90)	1(0.70)
Hemoptysis	58(19.73)	9(3.06)	11(7.69)	2(1.40)
Productive cough	49(16.67)	2(0.68)	16(11.19)	1(0.70)
Upper respiratory tract infection	33(11.22)	0	3(2.10)	0
Lung infection	28(9.52)	12(4.08)	9(6.29)	3(2.10)
Respiratory failure	10(3.40)	10(3.40)	3(2.10)	3(2.10)
Hypertension	198(67.35)	40(13.61)	23(16.08)	0
Sinus tachycardia	105(35.71)	0	47(32.87)	0
QT interval prolonged	77(26.19)	7(2.38)	27(18.88)	2(1.40)
Hand and foot skin reaction	128(43.54)	11(3.74)	13(9.09)	0

AEs	Anlotinib (N = 294) n %		Placebo (N=143) n%	
	All grades	Grade ≥3	All grades	Grade ≥3
Rash	35(11.90)	0	11(7.69)	1(0.70)
Chest pain	54(18.37)	1(0.34)	17(11.89)	3(2.10)
Flank pain	42(14.29)	0	11(7.69)	0
Pain in extremity	39(13.27)	0	16(11.19)	1(0.70)
Hypothyroidism	57(19.39)	1(0.34)	5(3.50)	0
Vertigo	33(11.22)	0	13(9.09)	0
Headache	32(10.88)	0	5(3.50)	0
Thyroid stimulating hormone increased	137(46.60)	1(0.34)	9(6.29)	0
Elevated triglycerides	126(42.86)	9(3.06)	34(23.78)	0
Elevated cholesterol	119(40.48)	0	20(13.99)	0
Gamma-glutamyltransferase increased	87(29.59)	13(4.42)	26(18.18)	9(6.29)
Blood bilirubin increased	76(25.85)	5(1.70)	21(14.69)	2(1.40)
Blood sodium decreased	66(22.45)	24(8.16)	12(8.39)	5(3.50)
Low density lipoprotein increased	60(20.41)	2(0.68)	11(7.69)	0
Lymphocyte count decreased	55(18.71)	14(4.76)	27(18.88)	8(5.59)
Aspartate aminotransferase increased (AST)	44(14.96)	3(1.02)	15(10.49)	0
Blood phosphorus decreased	31(10.54)	4(1.36)	10(6.99)	2(1.40)
Blood potassium decreased	31(10.54)	2(0.68)	7(4.90)	0
Platelet count decreased	30(10.20)	3(1.02)	6(4.20)	0
Lipase increased	17(5.78)	7(2.38)	2(1.40)	1(0.70)

Theoretical Rationale for the Study

The rationale is that combined inhibition of angiogenesis and PARP is dual. First of all, there is an opportunity for synergism; since anti-angiogenic agents can induce hypoxia in tumor [19], hypoxia will alter the DNA damage repair pathways, including the genetic instability caused by homologous recombination (HR) pathway [20], which increases the sensitivity of PARP inhibitors. This is called *contextual synthetic lethality*[21]. In addition, PARP-1 itself also plays a role in vascular inhibition and hypoxia response, transcribing and activating HIF-1α to respond to hypoxia [22].

Study Design and Study Plan

Overview

This is an open-label, single-center, prospective, single-arm phase II study to investigate the efficacy and safety of niraparib combined with anlotinib in the treatment of platinum-resistant recurrent ovarian cancer. In this study, 40 histopathologically diagnosed patients with high-grade serous ovarian, fallopian tube and primary peritoneal cancer were treated with niraparib plus anlotinib in patients who underwent first-line chemotherapy or above and had a recurrence of

platinum-resistant chemotherapy (the time of tumor progression of the last platinum-containing chemotherapy < 6 months).

Study Design Flow Chart

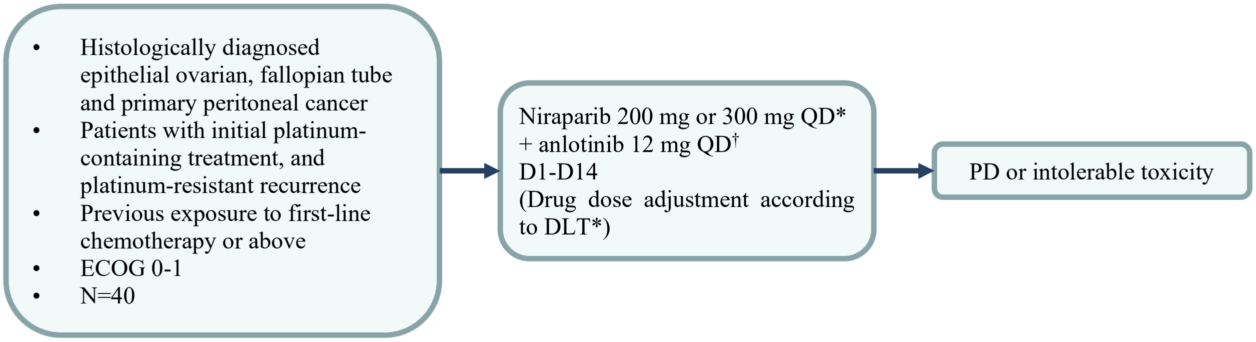

* The starting dose and dose reduction regimen of niraparib are as indicated in 4.3 dose modification of study drug

† Due to toxicities observed in the first 23 patients, the initial dose was lowered to 10mg QD on November 25, 2020 through protocol amendment.

Primary Objective

- To explore the anti-cancer activity of niraparib in combination with anlotinib for the treatment of platinum-resistant recurrent ovarian cancer.

Secondary Objectives

– To evaluate safety data of niraparib in combination with anlotinib.

– To evaluate PFS, platinum-free interval, and duration of response (DOR) of patients with platinum-resistant recurrent ovarian cancer treated with niraparib in combination with anlotinib.

– To evaluate the anti-cancer activity of niraparib in combination with anlotinib in patients with platinum-resistant recurrent ovarian cancer in various groups with *BRCA* mutation status (Yes or No) as a stratification factor.

Inclusion Criteria

1. Patients who understand the trial process, and have signed the informed consent form, and agree to participate in the study
2. Women aged 18 to 70 years (inclusive)
3. Histologically diagnosed epithelial ovarian, fallopian tube and primary peritoneal cancer
4. Patients who have the disease recurrence within 6 months after the end of the latest platinum-containing chemotherapy, that is, platinum resistance relapsed

5. Life expectancy>16 weeks
6. ECOG PS of 0-1
7. Patients who agree to take blood samples for g*BRCA* mutations
8. Patients who can provide formalin-fixed, paraffin-embedded tumor tissue samples for s*BRCA* and homologous recombination repair-related genes detection (optional)
9. Organ function is in good condition, including:
   - Neutrophil count ≥ 1,500/μL
   - Platelet count ≥100,000/μL
   - Haemoglobin ≥ 9 g/dL
   - Serum creatinine ≤ 1.5 × upper limit of normal (ULN), or creatinine clearance rate ≥ 60 mL/min (calculated according to Cockcroft-Gault formula)
   - Total bilirubin ≤ 1.5 × ULN or direct bilirubin ≤ 1.0 × ULN
   - AST and ALT ≤ 2.5 × ULN, or ≤ 5 × ULN in the presence of hepatic metastasis.
10. Only women of childbearing potential who have a negative pregnancy test result at enrollment, and promise to adopt effective contraception or be abstinent from the start to the end of the study and within 3 months after the last dose of the study drug can be enrolled into the study. Or females of non-childbearing potential may be enrolled in the study, defined as follows:
    - A female who has been surgically sterilized (hysterectomy, bilateral oophorectomy or bilateral salpingectomy); or
    - A female aged 60 years or older; or
    - A female, aged ≥40 years and <60 years, with a period of menolipsis for 12 months or longer, whose follicle-stimulating hormone test results are within the post-menopause reference range of the study institution.
11. Patients who are capable of complying with the protocol
12. Any previous chemotherapy-induced toxic and side effect of the patient has restored to CTCAE grade ≤1 or the baseline level, except for CTCAE grade ≤2 symptomatically stable sensory neuropathy or alopecia.
13. There is at least one measurable lesion before enrollment (according to RECIST 1.1);
14. The previous chemotherapy, radiotherapy, targeted therapy, immunotherapy or other anti-cancer treatments should be completed more than 4 weeks ago before the first dose of the study drug (more than 6 weeks for chemotherapy regimen containing mitomycin).

Exclusion Criteria

1. Have previously received any PARP inhibitor treatment
2. People who are known to be allergic to niraparib or active or inactive ingredients of drugs with similar chemical structure
3. People who are known to be allergic to anlotinib or active or inactive ingredients of drugs with similar chemical structure
4. Symptomatic, uncontrolled brain or leptomeningeal metastases. No imaging scans are required to confirm no brain metastases; patients with spinal cord compression may still be considered for enrollment if they have been treated with targeted therapy with evidence of clinical stabilization of the disease for at least > 28 days (metastases to central nervous system that are under control must have been treated with therapies such as radiotherapy or chemotherapy at least 1 month prior to enrollment; patients may not develop new symptoms associated with central nervous system lesions or symptoms suggestive of PD, and patients either take a stable dose of hormone or do not need to take hormone)
5. Patients who have underwent a major surgery within 3 weeks prior to the start of the study, or have any surgical effects that have not been recovered after surgery, or have received chemotherapy
6. Patients who have received palliative radiotherapy of > 20% bone marrow 1 week before enrollment
7. Patients who have invasive cancer other than ovarian cancer (except fully treated basal or squamous cell carcinomas of the skin) within 2 years before enrollment
8. Patients with central lung squamous cell carcinoma or at risk for massive hemoptysis (such as bronchiectasis, untreated tuberculosis)
9. Patients who have been previously or are currently diagnosed with myelodysplastic syndrome (MDS) or acute myeloid leukemia (AML)
10. Patients who have severe or uncontrolled diseases, including but not limited to:
    - Uncontrolled nausea and vomiting, inability to swallow the study drugs, or any gastrointestinal diseases that may intervene with and influence drug absorption and metabolism
    - Active viral infections such as human immunodeficiency virus, hepatitis B, hepatitis C infection, etc.
    - Uncontrolled grand mal seizure, unstable spinal cord compression, superior vena cava syndrome or other mental disorders influencing subjects to sign informed

consent forms

- Immunodeficiency (except for splenectomy), or other diseases which, as believed by investigators, can expose the patient to high-risk toxicity.

11. Patients with history of bleeding or thrombosis:
    (1) Any CTCAE Grade 2 bleeding event within 3 months prior to screening, or CTCAE Grade 3 and higher bleeding event within 6 months prior to screening
    (2) Gastrointestinal hemorrhage within 6 months prior to screening or clear tendency of gastrointestinal hemorrhage. For example, esophageal varices with risk of bleeding, focal active ulcer lesion, or fecal occult blood positive (++ and above)
    (3) Active bleeding or abnormal coagulation function, bleeding tendency or being treated with thrombolysis or anticoagulation
    (4) Anticoagulation therapy with an agent such as warfarin or heparin is required
    (5) Chronic anti-platelet therapy (such as aspirin, clopidogrel) is required
    (6) Thrombotic or embolic events within the past 6 months such as cerebrovascular accident (including transient ischemic attack), pulmonary embolism
12. Patients with history of serious cardiovascular diseases:
    (1) NYHA (New York Heart Association) Grade 3 and 4 congestive cardiac failure
    (2) Unstable angina pectoris or newly diagnosed angina pectoris or myocardial infarction within 12 months prior to screening
    (3) Arrhythmia requiring medications other than β-blockers or digoxin
    (4) Valvular heart disease of CTCAE grade ≥ 2
    (5) Hypertension inadequately controlled by drugs (systolic pressure >150 mmHg or diastolic pressure >100 mmHg)
13. The other abnormal laboratory test:
    - Hyponatremia (sodium < 130 mmol/L); baseline serum potassium < 3.5 mmol/L (serum potassium may be recovered above this level with a potassium supplement prior to study enrollment)
    - Abnormal thyroid function, which cannot be maintained within normal range with drugs
14. Patients with previous or current disease, treatment, or laboratory abnormality that may interfere with study results and influence them participating in the study, or patients not suitable for participation in the study at the investigator's discretion; patients are not

allowed to receive platelet or red blood cell transfusions within 4 weeks prior to study drug treatment

15. Patients who are pregnant or nursing, or who plan to become pregnant during study treatment
16. Corrected QT interval (QTc) > 450 msec; if a patient has prolonged QTc interval, but the investigator assesses that the prolongation is due to a pacemaker (and no other cardiac abnormalities), it is determined whether the patient is eligible for enrollment of the study by discussion with the investigator.

Patient Withdrawal and Replacement

End of treatment

Patients may discontinue study treatment at any time. Specific causes of the discontinuation include:

- Serious or life-threatening AEs
- Risk to the patient as judged by the investigator
- A critical protocol deviation as judged by the investigator
- Patient's decision
- Pregnancy
- PD
- If the clinical study continues, the investigator will predict the possible conditions or events that can jeopardize the patient.
- Patients are diagnosed as MDS or AML (as confirmed by hematologists)

Withdraw from the study

If the investigator removes the patient from the study, or the patient refuses to continue the study, a final assessment should be performed for the patient's disease status before any treatment intervention. These results, together with a description of causes for discontinuation, must be recorded in CRF.

Patients who discontinue treatment will continue to receive follow-up evaluation as part of the study unless they discontinue the study due to one of the following events:

- Withdrawal of consent
- Lost to follow-up
- Death from any cause

- Termination of study

When a subject discontinues study drug or withdraws from the study, the End of Treatment Visit (EOT) should be completed. Every effort should be made to contact the patients who are prematurely withdrawn and reluctant to return the site for the EOT. The investigator should inquire about the reasons why the patients are prematurely withdrawn and ask them to return all unused drugs and supplies for the study, and to complete the EOT, follow up and treat any unresolved AEs.

Study Management

Study Restrictions

Granulocyte colony-stimulating factor (GCSF)

Prophylactic cytokines (granulocyte colony-stimulating factor [GCSF]) should not be used in the first cycle of the study, but may be used in subsequent cycles according to local guidelines.

P-glycoprotein substrates

Niraparib has limited interaction with other drugs and has the potential to induce CYP1A2. Therefore, sensitive substrates of CYP1A2 (Alosetron, Caffeine, Duloxetine, Melatonin, Ramelteon, Tacrine, Tizanidine) should be taken with caution. Niraparib has the safety risk of thrombocytopenia, and patients are therefore advised to use anticoagulants and antiplatelet agents with caution.

Other anti-cancer therapy

Other anti-cancer therapies (including Chinese herbal medicines with anti-cancer effect) will not be allowed for any patient during the study treatment. This restriction no longer applies if the patient discontinues study treatment. In the context of absence of PD, palliative radiotherapy for mild pain metastases that existed before enrollment, could not be relieved by local or systemic application of analgesics will be allowed (patients who receive palliative radiotherapy for the pelvic cavity and/or over 20% of bone marrow area three weeks prior to enrollment will not be allowed).

Vaccine

When using conventional chemotherapy drugs, live-virus and bacterial vaccines were observed to increase the risk of infection. The effect of niraparib has not yet been clear, so patients are not allowed to be inoculated with live-virus/bacterial vaccines in this study. All patients will be informed of not being allowed for inoculation of live-virus/bacterial vaccines from the time of consent to the trial until at least 30 days after the end of treatment.

Contraception

Contraception guidelines

Patients with active sexual behavior and of childbearing potential and their partners must take two effective contraceptive measures from the start to the end of the study and within 3 months after the last dose of the study drugs. Condoms (male or female condoms, with or without spermicides) can be used as a form of birth control.

1. Acceptable non-hormonal contraceptive measures include:
   - Complete abstinence. Abstinence should be performed throughout the study, from your signing the informed consent form (ICF) to 90 days after the last dose of study drugs
   - For male sexual partners who have underwent vasectomy and use male condom containing spermicides. (You will need to confirm that your spouse has had sperm tested after a vasectomy and is confirmed to be sterile)
   - Fallopian tube obstruction plus male condom containing spermicides
   - Intrauterine device (IUD) plus male condom containing spermicides
2. Acceptable hormone methods include:
   - Etonogestrel implants (e.g., Implanon, Norplan) plus male condom containing spermicides
   - Normal and low-dose oral contraceptive pill plus male condom containing spermicides
   - Norelgestromin / ethinyl estradiol (EE) transdermal delivery system plus male condom containing spermicides
   - Intravaginal device (e.g., EE and etonogestrel) plus male condom containing spermicides

You should discuss with your doctor which contraception complies with the requirements.

No need for contraception

Patients must be postmenopausal, have no menstruation for more than 1 year, be surgically sterilized, be willing to use appropriate contraceptive measures to prevent pregnancy, or agree to complete abstinence within 3 months after the last dose of study treatment.

Blood donation

Patients should not donate blood during the study or within 90 days after the last dose of study treatment.

Schedule of Activities

See Section 7 Schedule of Activities.

Schedule of Visits

Screening process/enrollment (Visit 1, Day -28 to Day -1)

All subjects must sign and date the ICF before any study-specific screening procedure. Pre-enrollment screening test and evaluation will be used to determine the eligibility of patients to be included in the study. All screening tests must be completed within 28 days prior to enrollment, including the following:

1. Demographics, including past treatment records
2. Vital signs: body temperature, blood pressure, pulse rate, and respiratory rate
3. Physical examination, height and weight
4. Inquiry of prior and concomitant medication
5. ECOG PS score
6. Complete blood count (CBC) test and platelet count
7. Serum chemistry: ALT/SGPT, AST/SGOT, alkaline phosphatase, total bilirubin, creatinine clearance (calculated using Cockcroft formula) or creatinine, albumin, and electrolytes
8. Coagulation tests
9. Serum CA-125
10. Thyroid function test (TSH, free T3, free T4)
11. Urinalysis
12. 12-lead ECG
13. Serum or urine pregnancy test (within 3 days prior to the first dose of the study drugs) (only for women of childbearing potential)
14. RECIST 1.1 assessment: Abdomen/pelvic cavity and other clinically indicated sites

Once all screening tests are completed and the test results are reported, the investigator or his/her designated personnel will register the patient in the study.

During the study

The imaging examinations (enhanced CT scan or MRI scan) will be performed at treatment baseline, Week 9, Week 18, and Week 27, respectively, and will be independently read and reviewed by two radiologists from the blinded independent central review (BICR). Patients can continue treatment as long as there are no intolerable toxic and side effects, the disease is stable, or the treatment is effective. During the implementation of this study, the following items will be evaluated:

1. Physical examination (including vital signs), requiring retest of blood pressure every 10 minutes
2. ECOG PS score
3. Concomitant medication
4. Laboratory tests should be performed during the first cycle, and CBC should be performed on C1D1, C1D8 and C1D15, and then once per cycle
5. Circulating tumor DNA (ctDNA), tested weekly during the first cycle and every cycle thereafter; serum CA-125 should be tested every cycle
6. Coagulation test every cycle
7. Thyroid function test every 2 cycles
8. Urinalysis every cycle
9. 12-lead ECG every cycle
10. Monitoring of AEs
11. Whole blood specimen collection: The specimens are sent to the central laboratory designated by the investigator for g*BRCA* mutation detection. For patients who have been tested for g*BRCA* in the past, whether the previous test results can be directly used for study records is determined after discussion with the investigator.
12. Collection of formalin-fixed, paraffin-embedded tumor tissue samples: The samples are sent to the central laboratory designated by the investigator for testing of the HRD status or HRR-related genes (optional)
13. If the patient is diagnosed as MDS/AML during the study, bone marrow aspiration and biopsy will be completed, and the whole blood samples will be collected for molecular and cytogenetic analysis

Withdrawal from treatment for any reason (Visit is completed within 7 days after the last dose of study drug)

1. Vital signs

2. Physical examination, height and weight
3. Laboratory test
4. Coagulation function test
5. Thyroid function test
6. Urinalysis
7. 12-lead ECG
8. Monitoring of AEs

Continuous assessment

34. AEs will be monitored for 30 days after the last dose.
35. Subjects will receive survival follow-up after discontinuation of study treatment every 90 days (± 7 days) to collect the information of new malignant tumors.
36. Information (including commencement date of subsequent first anti-cancer treatment) of first new anti-cancer treatment (including chemotherapy) is collected after the end of study treatment every 90 days (± 7 days).

Unscheduled assessments

1. Monitoring of SAEs
   – If, at any time after study completion, the investigator identifies a study drug-related SAE, the investigator should report the SAE to the sponsor's pharmacovigilance department within 24 hours of learning of the SAE
2. CBC
   – CBC is tested weekly at any stage of the study if dose interruption or adjustment is required due to hematologic toxicities until the AE resolves. To ensure the safety of the adjusted dose, CBC should continue to be tested once a week, and the dose can be continued after 4 weeks of monitoring.
3. Chest CT or MRI
   – It is not necessary for repeating chest radiography if chest CT or MRI is clear at screening and there are no chest lesions or clinical indications; otherwise, chest radiography will be performed concurrently with RECIST.
4. Bone marrow aspiration and biopsy
   – Bone marrow aspiration and biopsy must be completed by a local hematologist for any suspected cases of MDS/AML that are reported while the patients are being treated or undergoing post-treatment evaluation. As long as the study site receives

a copy of the report of the hematologist and classifies it according to the World Health Organization standard, it will be a test of the treatment process. The study site must maintain a copy of the report and the patient's study file.

IMPs

Anlotinib

Management

The study drug should be stored in study sites as required in local regulations, which is safely stored with controlled and recorded temperature, and not available to unauthorized personnel. The investigator should ensure that the study drug is dispensed to the subject only and recycled as required. The study drug must be dispensed by authorized personnel at the study site in accordance with local regulations. Its receiving, distribution, storage, return and destruction must be correctly recorded.

Subjects take anlotinib hydrochloride capsules orally by themselves every morning before meals until PD or intolerance. They must show good compliance throughout the study. If the number of returned tablets does not match the expected number, it will be discussed with the subject and appropriate dose supplement will be conducted. At study drug return, compliance will be assessed based on drug count and subject diary. At each visit, drugs dispensed or returned to the sponsor are recorded for drug count and verification.

Packaging, labeling and storage

Anlotinib hydrochloride capsules will be packaged in aluminum foil and be available at dosage forms of 10 mg and 8 mg in this study. All study drugs will be numbered according to the requirements of Good Manufacturing Practice (GMP).

The investigator shall ensure that the storage conditions (temperature, light, and humidity) of the study drug are consistent with those determined by the drug manufacturer.

Anlotinib hydrochloride capsules should be stored below 25°C in sealed condition and protected from light. If there are temperature excursions or concerns regarding the quality and appearance of the study drug, isolate and suspend distribution of the study drug, and immediately contact the drug manufacturer.

Handling and destruction

Investigators should accurately record all the study drugs dispensed, including the date and quantity of distribution and recovery for each subject. After the end of the study, unused study drugs and empty bottles should be recycled and returned to the drug manufacturer for unified destruction.

Niraparib

Characteristics

Niraparib is an oral, potent, highly selective PARP-1 and PARP-2 inhibitor.

Management

Niraparib will be administered at a fixed dose (based on Phase I clinical trials), depending on the cohort of patients and not on body weight or body surface area. Each dose should be swallowed completely without chewing. It is allowed to swallow the drug with water. Patients should take the medicine at approximately the same time each day prior to bedtime and record this information in the patient diary regarding their use of study drugs. The actual number of tablets taken by the patient must be calculated according to the number of tablets dispensed and returned.

Patients must be instructed to return unused study drugs and empty packages back to the study site, at each visit, discontinuation or end of treatment. At each visit, drugs dispensed or returned to the sponsor are recorded for drug count and verification. The site staff must ensure that appropriate doses are used for each study drug.

All study drugs for treatment must be stored below 25 °C in sealed condition in accordance with manufacturer's instructions. They will be placed in access control places with locks that meet storage conditions and comprehensive safety precautions, as required by drug manufacturer.

Potential risk of niraparib

For information of AEs observed to date, please refer to the Investigator's Brochure.

MDS and AML have been observed in patients treated with PARP inhibitor, Olaparib. Given their common mechanism of action, MDS and AML are a potential risk for patients treated with niraparib.

Packaging and labeling

Niraparib 100 mg capsules are packaged with aluminum-plastic blisters, the box label text should include drug name, strength, package, sponsor's name, batch No., shelf life, clinical protocol No., administration and so on.

Handling and destruction

According to local regulations, if allowed, niraparib will be recycled to the drug manufacturer for destruction.

Prior and concomitant medications

Information on all concomitant medications during the study (generic name of drug, administration purpose, administration dose and time, etc.) must be detailed in the case report form (CRF). Treatment with other clinical investigational drugs will be prohibited during the study period.

Prohibited concomitant therapies:

(1) Radiotherapy for target lesion within 4 weeks prior to initiation of study drug treatment.

(2) During the treatment with the study drug, rifampicin (and its analogs), hypericum perforatum or other Chinese patent medicines (including, but not limited to, Xiaoaiping, Kanglaite, cinobufagin, elemene and Delisheng Injection) are not allowed to be co-administered.

(3) Anticoagulation therapy such as warfarin or heparin is not allowed when the subjects are administrated with the study drug.

(4) Other chemotherapy, hormone therapy, immunotherapy, therapeutic radiation, experimental anticancer drugs, or loco-regional therapy are not allowed while the subject is using the study drug. Subjects can continue to receive hormone replacement therapy.

Other restrictions and precautions.

Since anlotinib is mainly metabolized by CYP1A2 and CYP3A4/5, CYP3A4/5 inducers (rifampicin, rifabutin, rifapentine, dexamethasone, phenytoin, carbamazepine or phenobarbital, etc.) and CYP1A2 inducers (montelukast, omeprazole, moracizine, etc.) may accelerate the metabolism of anlotinib and reduce the blood concentration of anlotinib. Potent CYP3A4/5 inhibitors (ketoconazole, itraconazole, clarithromycin, voriconazole, telithromycin, saquinavir, ritonavir, etc.) and potent CYP1A2 inhibitors (ciprofloxacin, enoxacin and fluvoxamine) may slow down the metabolism of anlotinib and increase the blood concentration of anlotinib.

Anlotinib has moderate inhibitory effects on CYP3A4, CYP2B6, CYP2C8, CYP2C9 and CYP2C19 (IC_{50} within the range of 1-10 μM), but has no significant induction effect on CYP1A2, CYP2B6 and CYP3A4. Concomitant use of anlotinib with narrow therapeutic range drugs metabolized by these enzymes, such as alfentanil and ergotamine metabolized by CYP3A4, warfarin metabolized by CYP2C9, should be avoided.

The interaction between niraparib and other drugs is limited. Please refer to Appendix 2.

Table 7: **CYP3A4** inhibitors and inducers

CYP3A4 inhibitors	CYP3A4 inducers
HIV antivirus drugs<br>□ Indinavir<br>□ Nelfinavir<br>□ Ritonavir<br>□ Saquinavir	HIV antivirus drugs<br>□ Efavirenz<br>□ Nevirapine
Macrolides antibiotics<br>□ Clarithromycin<br>□ Erythromycin	Antiepileptic drugs<br>□ Carbamazepine<br>□ Phenytoin

CYP3A4 inhibitors	CYP3A4 inducers
Telithromycin	Phenobarbital
Calcium channel blockers<br>Diltiazem<br>Verapamil	Antibiotics<br>Rifabutin<br>Rifampicin
Azole antifungals<br>Fluconazole<br>Ketoconazole<br>Itraconazole	Others<br>Modafinil<br>Troglitazone<br>St. John’s wort
Others<br>Aprepitant<br>Nefazodone	

Dose modification of study drugs

The starting dose for the combination of niraparib and anlotinib in Phase II Study is determined by Phase I Study.

Niraparib

Niraparib should be orally taken before breakfast once daily for 3 weeks (a cycle). The drug should be continued until PD or intolerable adverse reactions. If a dose is missed during medication, and it is confirmed that the time from the next dose is less than 12 hours, skip the missed dose and take the next dose.

Starting dose

The starting dose is determined based on the subject's baseline body weight (but the dose adjustment after study initiation will not be based on body weight changes):

The starting dose is 300 mg (three 100 mg capsules) for the patient with baseline body weight of ≥ 77 kg.

The starting dose is 200 mg (two 100 mg capsules) for the patient with baseline body weight of < 77 kg.

Dose adjustment

During the use of niraparib, the adverse reactions should be closely monitored and the drug adjusted according to the adverse reactions so that the patients can tolerate the treatment. Adverse reactions caused by this product can be managed by symptomatic treatment, drug suspension and/or dose adjustment, etc. According to the degree of adverse reactions, it is recommended to adjust the dose under the guidance of the physician. If the dosage of 100 mg qd is still not tolerated, the discontinuation of drug is considered.

Dose interruptions and/or reductions can be implemented at any time for any grade of intolerable toxicity, but it is necessary to judge which drug and reduction strategy should be used in combination with the type of symptoms.

For any AEs of CTCAE (V.5) grade 3 or 4, the treatment must be interrupted. If the toxicity is appropriately reduced to baseline or grade 1 or lower within 28 days of interruption, the patient may restart the treatment, but dose levels are adjusted according to the following table.

If the AE of a similar or higher grade reoccurs, the treatment should be interrupted again, and the dose must be further reduced after it has been resolved.

If the toxicity has not been completely relieved or resolved to CTCAE grade 1 over 28 days of interruption, patients must discontinue the treatment.

Due to hematologic toxicities, dose interruption/adjustment criteria will be based on blood counts, which are listed in Table 8.

If hematologic toxicities are not resolved to the prescribed level within 4 weeks (28 days) after dose interruption, and/or the patient has one dose reduction (minimum dose: niraparib 100 mg/day), the patient must permanently discontinue niraparib treatment.

Table 8: Dose adjustment of ZL-2306 (Niraparib) due to hematologic toxicities

Results	Dose adjustment
Platelet count<100,000 /μL[(1)]	Suspend the study until platelet count ≥ 100,000/μL, and monitor CBC weekly until recovery.<br>Restart the treatment at the same dose or at a reduced dose based on clinical judgment.<br>If the platelet count is still not recovered to an acceptable level within consecutive 28 days of interruption or when the dose has been reduced to the lowest level (100 mg QD), discontinue the drug.
Platelet < 100,000/μL| recurs [(1)]	Suspend the study until platelet count ≥ 100,000/μL, and monitor CBC weekly until recovery.<br>Restart the treatment at a reduced dose.<br>If the platelet count is still not recovered to an acceptable level within consecutive 28 days of interruption or when the dose has been reduced to the lowest level (100 mg QD), discontinue the drug.
Neutrophil count <1.0 × 10^9/L	Suspend the study and observe blood cell count weekly for up to 28 days until neutrophil count recovers to ≥ 1.5×10^9/L.<br>The drug must be resumed at a reduced dose.<br>If the neutrophil count is still not recovered to an acceptable level within consecutive 28 days of interruption or when the dose has been reduced to the lowest level (100 mg QD), discontinue the drug.
Haemoglobin < 80 g/L	Suspend the study and observe blood cell count weekly for up to 28 days until haemoglobin recovers to ≥ 90 g/L.<br>ZL-2306 (niraparib) must be resumed at a reduced dose.

Results	Dose adjustment
	If the haemoglobin is still not recovered to an acceptable level within consecutive 28 days of interruption or when the dose has been reduced to the lowest level (100 mg QD), discontinue the drug.
Hematologic AEs require blood transfusion or supportive therapy with haemopoietic growth factor.	If the patient's platelet count is ≤ 10×10^9/L, platelet transfusion should be considered. If there are other coexisting risk factors, such as anticoagulants or antiplatelet drugs, consider suspending these drugs and/or transfusing blood until platelet count reaches a higher level.<br>The drug must be resumed at a reduced dose.
Subjects confirmed with MDS or AML	Permanently discontinue the drug

Abbreviations: CBC = complete blood count

(1) If the platelet count is not recovered to ≥ 100,000/L within 28 days of dose interruption, the patient should stop medication.

During any stage of the study, if dose interruption or adjustment is required due to hematologic toxicities, CBC should be monitored weekly until AE remission. In order to ensure dose safety for restarting the treatment, CBC should still be monitored weekly. After the condition stabilizes during 4-week monitoring, the CBC can be resumed to be retested once every 4 weeks.

For any patient who needs transfusion of platelets or red blood cells (one or more units) or haemopoietic growth factor support, if the study treatment is resumed, the dose must be reduced after resumption.

Patients who require more than 1 blood transfusion or whose treatment-related hematologic toxicities are not resolved to CTCAE grade 1 or below after 4 weeks must be referred to a hematologist for further evaluation.

For patients who need surgical treatment midway, it is necessary to consider withdrawing from treatment.

All dose interruptions and reductions (including any missed doses) and reasons for reduction/interruption should be documented.

For patients starting from 200 mg, it may be increased to 300 mg if there is no dose interruption or reduction caused by adverse reactions in the first two cycles of treatment.

After reduction of study drugs from starting dose of 300 mg QD to 200 mg QD, the dose escalation back to 300 mg QD may be considered if it is well tolerated during at least two treatment cycles. After reduction of study drugs from 200 mg QD to 100 mg QD, the dose escalation back to 200 mg QD may be considered if it is well tolerated during at least one treatment cycle.

Anlotinib

Anlotinib hydrochloride should be orally taken before breakfast once daily (on for 2 weeks and off for 1 week), with a cycle of 3 weeks (21 days), until PD or intolerable adverse reactions. If a

dose is missed during medication, and it is confirmed that the time from the next dose is less than 12 hours, skip the missed dose and take the next dose.

Dose adjustment

During the use of anlotinib, the adverse reactions should be closely monitored and the drug adjusted according to the adverse reactions so that the patients can tolerate the treatment. Adverse reactions caused by this product can be managed by symptomatic treatment, drug suspension and/or dose adjustment, etc. According to the degree of adverse reactions, the dose is adjusted under the guidance of the investigator: dose adjustment: 8 mg, once a day, on for 2 weeks and off for 1 week;

The following table presents the recommended regimen for delaying administration time and/or changing dose level in the event of the study drug-related toxicity. When patients develop toxicities such as **function liver abnormal, bleeding, proteinuria, and platelet count decreased**, please perform with reference to the requirements of subsequent separate tables.

Grade of adverse reaction NCI CTC AE Version 4.0	Administration time	Dose adjustment
Grade 0-2	Administrate on time	Unchanged
Grade 3	Delayed administration until resolution to grade <2 #	Reduce by one dose level
Grade 4	Delayed administration until resolution to grade <2 #	Reduce by one dose level. According to the treatment, the investigator judges that permanent termination of treatment may be considered.

#: If it still has not recovered after delayed administration for 2 weeks, treatment should be permanently terminated.

The following table shows the recommended regimen for delaying administration time and/or changing dose level when function liver abnormal occurs (ALT, AST or total bilirubin increased).

AE grade	Dose adjustment regimen	Handling recommendation
Grade 1	Maintain original dose	Have follow-up as scheduled
Grade 2 (normal at baseline)	Delay administration, if it is recovered to grade < 2 within 2 weeks, reduce a dose level	Give active hepatoprotective treatment and closely monitor liver function, once a week
Grade 2 (abnormal at baseline)	Maintain original dose	Give active hepatoprotective treatment and closely monitor liver function, once a week
Grade 3	Delay administration, if it is recovered to grade < 2 within 2 weeks, reduce a dose level	Give active hepatoprotective treatment and closely monitor liver function, twice a week; until the toxicity is resolved to grade < 2 or justified

AE grade	Dose adjustment regimen	Handling recommendation
Grade 4	Permanently terminate treatment	Give active hepatoprotective treatment and closely monitor liver function, once to twice a week; until the toxicity is resolved to grade < 2 or justified

The following table shows the recommended regimen for delaying administration time and/or changing dose level when proteinuria occurs

AE grade	Dose adjustment regimen	Handling recommendation
Grade 1: Urinalysis indicates urine protein positive (+) or 24 h urinary protein quantitation result of < 1.0 g	Maintain original dose	Have visit as scheduled
Grade 2: Urinalysis indicates urine protein positive (++), but 24 h urinary protein quantitation result of 1.0 g - 2.0 g (exclusive)	Maintain original dose	Give active treatment and monitor urinalysis (once a week); consult a nephrologist if necessary
Grade 2: Urinalysis indicates urine protein positive (++) or above, 24 h urinary protein quantitation result of 2.0 g - 3.5 g (exclusive)	Delay administration. If it is recovered to grade < 2 within 2 weeks, reduce a dose level for subsequent medication.	Give active treatment, consult a nephrologist if necessary, and the treatment must be terminated permanently in case of the third occurrence;
Grade 3: 24 h urinary protein quantitation result of ≥ 3.5 g	Delay administration. If it is recovered to grade < 2 within 2 weeks, reduce a dose level for subsequent medication	Give active treatment, consult a nephrologist if necessary, and the treatment must be terminated permanently in case of the third occurrence;

The following table shows the recommended regimen for delaying administration time and/or changing dose level when platelet count decreases

AE grade	Dose adjustment regimen	Handling recommendation
Grade 1: the platelet count is 100~75× 10^9/L	Maintain original dose	Have visit as scheduled
Grade 2: the platelet count is 75~50× 10^9/L	Delay administration, if it is recovered to grade < 2 within 1 week, maintain original dose	Retest the hematology once every 2 to 3 days; and recommend to have active treatment. Retest hematology once every week during the follow-up visit
	Delay administration, if it is recovered to grade < 2 within 2 weeks, reduce a dose level	Retest the hematology once every 2 to 3 days; and recommend to have active treatment. Retest hematology once every week during the follow-up visit

AE grade	Dose adjustment regimen	Handling recommendation
Grade 3: Platelet count 50~25× 10^9/L	Delay administration, if it is recovered to grade < 2 within 2 weeks, reduce a dose level	Retest the hematology once every 2 to 3 days; and recommend to have active treatment. Retest hematology once every week during the follow-up visit
Grade 4: Platelet count <25×10^9/L	Permanently terminate treatment	Retest the hematology every day until resolution to grade ≤ 2; infuse apheresis platelets and give active treatment.

The following table shows the recommended regimen for delaying administration time and/or changing dose level when the bleeding event occurs

AE grade	Dose adjustment regimen	Handling recommendation
Grade 1	Maintain original dose	Have follow-up as scheduled
Grade 2	Delay administration, if it is recovered to grade < 2 within 2 weeks, reduce a dose level	Give active treatment
Grade ≥3	Permanently terminate treatment	Emergency medical intervention

As previously described, in the ALTER0303 clinical trial, dose reduction (from 12 mg to 10 mg) occurred only in 25 patients (8.50%) of the anlotinib group, and the second reduction (from 10 mg to 8 mg) occurred in 3 patients (1.02%). The main AEs leading to dose reduction were hand and foot skin reaction, hypertension, diarrhea, anorexia, stomatitis, function liver abnormal, proteinuria, lipids blood increased, and asthenia. In the NOVA study, more than 5% of grade 3 and higher AEs for niraparib were thrombocytopenia (33.8%), anaemia (25.3%), neutropenia (19.6%), asthenia (8.2%) and hypertension (8.2%).

Specifically marked AE of circulatory system: hypertension. As hypertension is a common AE of anti-angiogenic agents and combined treatment is used the clinical trial, the hypertension AE in the niraparib study set will be separately described. Of the 384 patients in the niraparib study set of ovarian cancer treatment, 28 patients (7%) reported hypertension as a TEAE. Ten (3%) of 384 patients reported to have grade 3 and higher hypertension. The incidence of hypertension in this study set was lower than that in NOVA study, among which the incidence in the niraparib group was 19%, and the incidence of grade 3 and higher events was 8%. However, the incidence of patient-exposure years (PEY) of hypertension in NOVA study (0.29) was similar to the result (0.30) reported in the study set. In the QUADRA study, 1 patient reported discontinuation of study treatment due to hypertension. Six (2%) patients interrupted the dose due to hypertension, and no patient reduced the dose due to hypertension. Before the start of the study, two cardiovascular physicians (deputy chief physician or above) will be designated as consultants in this project to manage the AE of blood pressure increased in the progress of the project. Subjects whose blood

pressure cannot be effectively controlled by antihypertensive drugs will be withdrawn from this study.

Hypotheses and Statistical Method

The ORR for standard chemotherapy in patients with platinum-resistant recurrent ovarian cancer is approximately 10–20% [23,24]. Assuming that the true objective response rate with anlotinib plus niraparib was 40%, a sample size of 36 patients was estimated to provide 80% power to reject the null hypothesis of an objective response rate ≤20% at a one-sided type-1 error rate of 5%. Assuming a 10% rate of loss to follow-up, a target sample size of 40 patients was planned.

Primary Endpoint:

To evaluate the ORR for niraparib in combination with anlotinib in patients with platinum-resistant recurrent ovarian cancer, i.e., the proportion of subjects achieving complete response (CR) and partial response (PR) in all patients.

Secondary Endpoint 1:

To evaluate safety data of niraparib in combination with anlotinib

Secondary Endpoint 2:

To evaluate the time to response (TTR), duration of response (DOR), overall response rate (ORR), and 24-week clinical benefit rate (CBR=CR+PR+SD) for niraparib in combination with anlotinib in patients with platinum-resistant recurrent ovarian cancer.

Secondary Endpoint 3:

To evaluate the anti-cancer activity of niraparib in combination with anlotinib in patients with platinum-resistant recurrent ovarian cancer in various groups with *BRCA* mutation status (Yes or No) as a stratification factor.

Tumor assessment

Therapeutic efficacy is assessed according to RECIST 1.1 for all measurable lesions. The assessment doctors are two independent senior radiologists regularly engaged for this study. They do not participate in other diagnosis and treatment activities of this study, and only perform imaging assessment. Tumor assessments for treatment response will be performed every 9 weeks until PD. All disease sites must be assessed using baseline assessment method. Confirmatory assessments of CR or PR must be performed at least 4 weeks after the initially documented response.

All tumor marker assessments must be performed by the same laboratory for each patient. All target lesions will be measured by the same imaging technique throughout the study. The same technique should be used for each patient' s assessment. The results should be recorded in the CRF. Imaging copies may be provided for review. Patients who receive at least 2 treatment cycles and

at least one disease assessment during the study, or patients with early progression, will be regarded as evaluable patients.

Safety Analysis

Trained study coordinators and/or data administrators will enter the information described in this section. All AEs will be coded using MedDRA terminology. In addition, a table of incidence of individual AEs coded by MedDRA terminology will be presented according to the following criteria: 1) Toxicity grade (seriousness), classified per the Common Terminology Criteria for Adverse Events (CTCAE) v5.0; 2) Determination of the relationship between AEs and treatment with the investigator.

All discontinuation of drug caused by SAEs and AEs, or deaths during trial or within 30 days after trial termination will be summarized per each patient.

Patient survival information and information of MDS/AML patients will be collected every 8 weeks from the start of treatment until 90 days after the end of the study.

Missing Data n/a

Missing data will not be imputed.

Interim Analysis n/a

Phase I patients are followed up to week 24 after the end of enrollment. If ORR value cannot reach 20%, the study will be terminated. Safety data will be continuously reviewed.

Ethical, Legal and Administrative Provisions

GCP

This clinical study shall be implemented in accordance with the *Declaration of Helsinki*, Good Clinical Practice in China and regulations of drug clinical trials.

Informed Consent

The investigator has the responsibility to explain to each patient the purpose, methods, benefits and potential risks of this clinical study. Signed ICF must be obtained from patients before conducting any study-specific procedures. Informed consent should be expressed in written form. The ICF must be dated and signed by the patient him/herself. For those patients who could not sign the ICF by themselves for any reasons, their parents, legal guardians, or protectors should sign the ICF. The original copy of signed ICF must be preserved and documented in the CRF and relevant trial source documents by the investigator.

By signing the ICF, the patient must also agree to allow the investigator, the drug approval authorities, and the auditor and (or) the monitor to check and verify the obtained source data and

materials related to the clinical study, and all parties mentioned above must comply with confidentiality statement.

Approval and Amendment of Protocol

After the final version of the study protocol is confirmed, any modifications to the protocol must be documented in detail and signed, and the version number and date should be also indicated.

The protocol amendment should involve the sponsors and the investigator. The amendment should not be implemented before obtaining the approval of the Ethics Committee.

Confidentiality and Data Protection of Subjects

The investigator must keep confidential the information about the study drug that is provided or disclosed for the clinical study cooperation, and such information can only be used with authorization.

Investigators are obligated to protect the privacy of patients participating in the clinical study. In all the documents, the identity of any patient in the clinical study will be coded by the patient number, while the patient's name, admission number should not show up. The investigator must appropriately keep name and address of clinical trial patients, as well as enrollment table corresponding to clinical study patient number. These enrollment tables will be strictly kept confidential and stored by the investigator.

Source Data Verification

The investigator must appropriately handle all data obtained during the clinical study to ensure rights and privacy of patients participating in the clinical study. The investigator must permit the monitor/auditor/inspector to consult and review required clinical study data to verify accuracy of original data and learn about study progress. If the original records cannot be verified, the investigator should agree to assist to further validation of data quality with them.

Quality Assurance, Review and Approval

The quality of all drugs and materials used in the clinical study must be controlled. The personnel authorized by investigator or related medical management agencies have the right to audit the clinical study in order to ensure the authenticity of the clinical study record data and comply with the provisions of the clinical study protocol. Patients participating in the clinical study will be informed of the audit, but the privacy and data of patients will be strictly protected.

Monitoring

The investigator assigns certain monitor to perform on-site monitoring. The monitors should operate in compliance with the company's standard operating procedure (SOP). They should perform regular visits from the start to the end of study.

The monitors will have access to relevant source data of the clinical study, and review CRF according to SOP to confirm information completeness, accuracy and consistency with original data.

CRFs, copies of lab tests and medical tests must be accessible to monitors, auditors and health authorities for review at any time. The monitors should review all CRFs and ICFs.

Safety Monitoring and Reporting

Definition of AEs

An adverse event is any untoward medical occurrence in a patient or subject administered a pharmaceutical product and which does not necessarily have to have a causal relationship with this treatment. Example of AEs include, but not limited to adverse signs (including abnormal findings in laboratory test) and symptoms, conditions that have a temporal relationship with use of study drug, regardless of causal relationship to the study drug. AEs include serious adverse events (SAEs) and non-serious AEs.

Definition of SAEs

A SAE is a serious adverse event that occurs during the course of a clinical trial and may include:

- Events leading to death;
- Life-threatening events (refers to an event in which the subject is at risk of death at the time of the event);
- Events requiring hospitalization or prolongation of hospitalization;
- Events that result in permanent or significant disability/ incapacity/ impairment of work;
- Events that result in congenital abnormality or birth defect;
- Other important medical event (defined as an event/events that may jeopardize the subject or may require intervention to prevent one of above listed outcomes).

The following scenarios **will not** be reported as SAEs:

- Any results, including death, caused by tumor progression as expected by investigator.
- Hospitalization or prolonged hospitalization due to economic issue or for purpose of reimbursement.

AE recording

The AEs collection and recording will be throughout the clinical trial, from the signing of the ICF to the end of the trial (30 days after the last dose of study drugs). AEs and SAEs that occur following the signing of the ICF but before the first dose should be recorded on CRF as pretreatment symptoms and signs, and not collected as AEs, but if they deteriorate during the trial,

such events should be recorded as AEs. PD events are efficacy criteria and therefore not considered as AEs. If AEs/SAEs are related to PD, these AEs/SAEs must be reported as required.

AE assessment

The investigator will evaluate each AE for the following categories:

- Severity
- Intensity
- Causal relationship

Severity and causality should be evaluated according to the International Conference on Harmonization, Technical Requirements for Clinical Safety Data Management (E2A Guidance). The intensity should be evaluated according to the Common Terminology Criteria for Adverse Events (CTCAE v.5).

Reporting of SAEs

Any SAEs occurring during the trial, regardless of causal relationship to the treatment, should be reported by following the SAE reporting procedures required by competent regulatory authorities or Independent Ethics Committee.

The investigator should:

(1) Immediately take appropriate medical measures, if necessary;

(2) Record SAEs in the AE Form of CRF, SAE Form and source documents.

(3) The investigator should immediately (within 24 hours of becoming aware of the event) send the singed and dated SAE Form to the following parties: A. Independent Ethics Committee, B. Drug Safety Department of the Sponsors (fax or email as follows), C. Provincial and China Food and Drug Administrations (report through network system or fax according to provincial bureau's provisions); and D. National Health and Family Planning Commission of the PRC.

Please send SAE reports to the public email box of Zai Lab's Drug Safety Department: saereporting@zailaboratory.com

Drug Safety Department, Chia Tai Tianqing Pharmaceutical Group Co., Ltd.

Contact person 1: Zai Lab’s Drug Safety Officer: +86 13501637451

Contact person 2: Zai Lab’s Drug Safety Officer: +86 13611624754

Contact person 3: Chia Tai Tianqing’s Drug Safety Officer:

Fixed-line telephone: +86 10 6505 3288-608

Fax: +86 10 65059826; +86 21 61632570

(4) Follow-up of SAEs will be pursued until they are recovered/resolved to the baseline level or clinically stable.

All AEs will be recorded from the signing of the ICF to 30 days after study treatment termination. The SAE that occurs after this 30-day period and attracts the attention of the investigator must be reported if, in the opinion of the investigator, it is causally associated with the study drug.

Follow-up of AEs

The investigator shall continue to follow up any AEs that are not resolved at the last visit of the patient in this study until there are no clinical indications. Subsequent follow-up may be excluded from the CRF, but the sponsors shall have the right to request the investigator to provide further information when deemed necessary.

Suspected unexpected serious adverse reactions (SUSARs)

For any serious AE in the study and unexpected (defined as any term not listed in the current Investigator's Brochure) AEs related to the study treatment, the additional reporting requirements are as follows. These types of reports are known as (SUSARs).

- If the SUSAR is a fatal or life-threatening, study treatment-related, unexpected AE, the management authority and Independent Ethics Committees (IECs) will be notified within 7 days of the investigator learning of the event. Information (cause of death, necropsy report, and hospital report) obtained from unscheduled follow-up should be reported within the next 8 days (15 days in total).
- If the SUSAR is not fatal or life-threatening, but is serious in other aspects, and is an unexpected AE related to study treatment, the regulatory authorities and IECs will be notified within 15 days after the investigator learning of the event.

This organization will also provide annual safety data updates to regulatory authorities and IECs. These updates will include information on SUSARs and other related safety information.

Pregnancy

Patients enrolled in this study are required for contraception. If a subject is found to be pregnant following administration, the study drug will be discontinued and the subject will be withdrawn from the trial. The investigator will immediately report any pregnancy event to the sponsors by phone or fax within 24 hours after learning of it, follow up the event until the end of pregnancy, and inform the sponsors of the pregnancy outcome. If the outcome of the pregnancy meets SAE criteria (e.g., spontaneous abortion or therapeutic abortion [with any detected congenital anomaly in fetus documented], stillbirth, neonatal death, or birth defect), it will be reported as SAE. The investigator should send the completed "SAE Reporting Form" to the sponsors (or designee) via phone or fax within 24 hours of becoming aware of the event.

IMPs

The investigator should be responsible for and take all measures for proper records, and ensure that the supply, treatment, storage, distribution and use of the study drug comply with the study protocol and any applicable laws and regulations.

Dispensing

The investigator agree that the study drug will be dispensed by personnel designated in the investigator's agreement or his/her qualified designated personnel, and the study drug is only available to subjects who have provided written ICFs and meet all inclusion criteria and the instructions set forth in the *Pharmacy Manual*.

Drug liability

The investigator or his/her designated personnel is responsible for completely recording the accurate distribution of study drug throughout the clinical study. Accountability records for study treatment include registration number, quantity and date of distribution, and quantity and date of return to the pharmacy (if applicable). Drugs returned to the pharmacy will be kept under the same conditions as those not dispensed, but will be marked as "returned" and stored separately from undispensed drugs.

All records of dispensing and responsibility should be kept according to the instructions of the sponsor-investigator.

Paper publication

The investigator will decide to submit the resulting paper for publication, or any work directly related to the study.

Schedule of Activities

Table 11. Schedule of Activities

Study period	Screening visit	Cycle 1			Cycle 2	Cycle 3	Subsequent cycles	EOT	Assessment after treatment
Day	(≤28 days)	1	8	15	1	1	1	Within 7 days after the last dose	30 days post-treatment
Sign the ICF	X								
Inclusion/exclusion criteria	X								
Demographics	X								
Weight and height	X							X	X
Vital signs	X	X	X	X	X	X	X	X	X
ECOG score[a]	X	X			X	X	X		X
Medical history and medication history	X								
Physical examination	X	X			X	X	X	X	X
Concomitant medication	X	X			X	X	X	X	X
Pregnancy test[b]	X								
Laboratory test[d]	X[c]	X[f]	X	X	X	X	X	X	
Coagulation function test	X[c]	X[f]			X	X	X	X	
Thyroid function test	X[c]	X[f]				Retest once every 2 cycles		X	
Urinalysis	X[c]	X[f]			X	X	X	X	
12-lead ECG	X	X			X	X	X	X	
Serum CA-125	X	X[f]			Retest once every cycle				
Peripheral blood (*BRCA* gene detection)		X							
Tumor assessment	X					X	Once every 3 cycles		
Monitoring of AEs		X				X	X	X	X[e]

Footnotes:

a. PS score of 0-1 (Please refer to Appendix -1 Eastern Cooperative Oncology Group (ECOG) Performance Status Score).

b. Female subjects of childbearing potential must have a serum or urine pregnancy test, which will be performed locally 3 days prior to study medication.

c. Laboratory assessments in the screening stage must be performed within 7 days prior to study medication.

d. Laboratory tests include absolute neutrophil count, platelet count, haemoglobin, serum creatinine, total bilirubin, aspartate aminotransferase, alanine aminotransferase, and electrolytes.

e. AEs should be monitored for 30 days after the last dose.

f. Repeat is not required if it is performed within 7 days prior to the first day of the study.

Reference

1. W. Chen, Zheng R, Baade PD, et al. Cancer statistics in China, 2015. CA Cancer J Clin 2016;66:115–132.
2. Fotopoulou C. Limitations to the use of carboplatin-based therapy in advanced ovarian cancer. European Journal of Cancer. Suppl. 2014; 12(2):13-16.
3. Gore ME, Fryatt I, Wiltshaw E, Dawson T. Treatment of relapsed carcinoma of the ovary with cisplatin or carboplatin following initial treatment with these compounds. Gynecologic Oncology. 1990; 36(2):207-211.
4. Markman M, Rothman R, Hakes T, et al. Second-line platinum therapy in patients with ovarian cancer previously treated with cisplatin. Journal of Clinical Oncology. 1991; 9(3):389-393.
5. Amé J-C, Spenlehauer C, de Murcia G. The PARP super-family. BioEssays. 2004; 26(8):882–93.
6. Kaelin WG, Jr. The concept of synthetic lethality in the context of anticancer therapy. Nature Review Cancer. 2005; 5(9):689-698.
7. Ledermann J, Harter P, Gourley C, et al. Olaparib maintenance therapy in patients with platinum-sensitive relapsed serous ovarian cancer: a preplanned retrospective analysis of outcomes by BRCA status in a randomised phase 2 trial. Lancet Oncology. 2014; 15(8):852-861.
8. Kaufman B, Shapira-Frommer R, Schmutzler RK, et al. Olaparib monotherapy in patients with advanced cancer and a germline BRCA1/2 mutation. Journal of Clinical Oncology. 2015; 33(3):244-250.
9. Ledermann J, Harter P, Gourley C, et al. Olaparib maintenance therapy in platinum-sensitive relapsed ovarian cancer. New England Journal of Medicine. 2012; 366(15):1382-1392.
10. Mirza MR, Monk BJ, Herrstedt J, Oza AM, Mahner S, Redondo A, et al. niraparib Maintenance Therapy in Platinum-Sensitive, Recurrent Ovarian Cancer. New England Journal of Medicine. 2016; October 7, online version.
11. Watkins JA, Irshad S, Grigoriadis A, Tutt AN. Genomic scars as biomarkers of homologous recombination deficiency and drug response in breast and ovarian cancers. Breast Cancer Res. 2014; 16 (3): 211.
12. Kaelin WG, Jr. The concept of synthetic lethality in the context of anticancer therapy. Nat Rev Cancer. 2005; 5 (9): 689-698.
13. Kathleen N Moore, et al. niraparib monotherapy for late-line treatment of ovarian cancer (QUADRA): a multicentre, open-label, single-arm, phase 2 trial. Lancet Oncol 2019 Published Online April 1, 2019 http://dx.doi.org/10.1016/S1470-

2045(19)30029-4

14. Radha V, Nambirajan S, Swarup G (March 1996). "Association of Lyn tyrosine kinase with the nuclear matrix and cell-cycle-dependent changes in matrix-associated tyrosine kinase activity". European Journal of Biochemistry. 236 (2): 352–9. doi:10.1111/j.1432-1033.1996.00352.x. PMID 8612602.

15. NCCN guidelines for ovarian cancer version 1 2019. https://www.nccn.org/store/login/login.aspx?ReturnURL=https://www.nccn.org/professionals/physician_gls/pdf/ovarian.pdf

16. J. F. Liu, W. T. Barry, M. Birrer, et al. Overall survival and updated progression-free survival outcomes in a randomized phase II study of combination cediranib and olaparib versus olaparib in relapsed platinum-sensitive ovarian cancer. Annals of Oncology 0: 1–7, 2019 doi:10.1093/annonc/mdz018 Published online 7 February 2019

17. Han B, et al. Effect of anlotinib as a third-line or further treatment on overall survival of patients with advanced non–small cell lung cancer: The ALTER 0303 phase 3 randomized clinical trial JAMA Oncology August 15, 2018

18. China Food and Drug Administration (CFDA) Catalog Set of Marketed Drugs http://202.96.26.102/index/detail/id/363

19. Ellis LM, Hicklin DJ. VEGF-targeted therapy: mechanisms of anti-tumour activity. Nat Rev Cancer. 2008 Aug;8(8):579.

20. Bristow RG, Hill RP. Hypoxia and metabolism. Hypoxia, DNA repair and genetic instability. Nat Rev Cancer. 2008 Mar;8(3):180-92.

21. Chan N, Bristow RG. "Contextual" synthetic lethality and/or loss of heterozygosity: tumor hypoxia and modification of DNA repair. Clin Cancer Res. 2010 Sep 15;16(18):4553-60.

22. Rohwer N, Zasada C, Kempa S, et al. The growing complexity of HIF-1α's role in tumorigenesis: DNA repair and beyond. Oncogene. 2013 Aug 1;32(31):3569-76.

23. J.-M. Ferrero, B. Weber， et al. Second-line chemotherapy with pegylated liposomal doxorubicin and carboplatin is highly effective in patients with advanced ovarian cancer in late relapse: a GINECO phase II trial. Annals of Oncology 18: 263–268, 2007

24. Parmar MK, Ledermann JA, et al. Paclitaxel plus platinum-based chemotherapy versus conventional platinum-based chemotherapy in women with relapsed ovarian cancer: the ICON4/AGO-OVAR-2.2 trial. Lancet. 2003 Jun 21;361(9375):2099-106.

Appendix

Appendix 1-Eastern Cooperative Oncology Group (ECOG) Performance Status Score

Description of physical status	Score
Completely normal, being able to perform work before illness without restriction	0
Not able to carry out heavy physical work, but be able to walk around and carry out light physical work such as light housework or office work	1
Can move around, capable of self-care, but unable to carry out any work-related activities, confined to bed for less than 50% of the day	2
Self-care can only be achieved to a limited extent, with more than 50% of the daytime spent in bed rest or chair sitting	3
Complete loss of activity of daily living, unable to achieve selfcare, confined to bed or wheelchair	4

Available at: http://ecog-acrin.org/resources/ecog-performance-status
