# Supplementary material for: A novel combination of niraparib and anlotinib in platinum-resistant ovarian cancer: Efficacy and safety results from the phase II, multi-center ANNIE study
Source: eClinicalMedicine. 2022 Nov 30;54:101767. doi: 10.1016/j.eclinm.2022.101767 (PMC9793276; doi:10.1016/j.eclinm.2022.101767)
Supplement: Captions for Supplementary Materials [file mmc2.docx]

**Captions for Supplementary Materials/Appendix**

Supplementary Materials:

Supplementary Table 1. Prior Antiangiogenic Treatments Received by Patients from the ANNIE Study (N=40)

Supplementary Table 2. Treatment-Emergent Adverse Events Documented in ANNIE (n=40)

Supplementary Figure 1. Post-hoc subgroup analyses of hazard ratios for progression-free survival (PFS)

Supplementary Figure 2. Kaplan-Meier curve for time to response (TTR) for the 20 patients who achieved an objective response

Supplementary Figure 3. Kaplan-Meier curve for duration of response (DOR) for the 20 patients who achieved an objective response

Supplementary Figure 4. Kaplan-Meier curve for platinum-free interval (PFI)

Supplementary Figure 5. Kaplan-Meier curves for progression-free survival (PFS) in subgroups with and without prior antiangiogenic treatment

Supplementary Figure 6. Kaplan-Meier curves for overall survival (OS) in subgroups with different numbers of prior lines of chemotherapy (post-hoc)

Supplementary Figure 7. Kaplan-Meier curves for overall survival (OS) in subgroups with and without prior antiangiogenic treatment (post-hoc)

Supplementary Figure 8. Exploratory analysis of the relationship between circulating tumour markers and prognosis

Manuscript Appendix:

ANNIE Study Protocol (Version 1.4)
